# Novel Asymmetric Iron Porphyrins for Photocatalytic CO_2_ Reduction to CH_4_

**DOI:** 10.1002/cssc.202500715

**Published:** 2025-07-17

**Authors:** Edelman José Espinoza‐Suárez, Akhmet Bekaliyev, Aranza Vital‐Grappin, Laura Velasco‐Garcia, Laia Subirats Valls, Carla Casadevall

**Affiliations:** ^1^ Department of Physical and Inorganic Chemistry University Rovira i Virgili (URV) C/ Marcel.lí Domingo, 1 Tarragona 43007 Spain; ^2^ Institute of Chemical Research of Catalonia (ICIQ) The Barcelona Institute of Science and Technology (BIST) Av. Països Catalans 16 Tarragona 43007 Spain

**Keywords:** artificial photosynthesis, CO_2_ reduction, CO_2_R‐to‐CH_4_, iron porphyrins, photocatalysis

## Abstract

Developing earth‐abundant transition metal catalysts for CO_2_ reduction is a promising approach for sustainable energy conversion. Here, the synthesis and photocatalytic activity of two novel asymmetric iron porphyrin complexes, namely iron 5‐(*N*‐benzyloxycarbonyl‐4‐aminophenyl)‐10,15,20‐tris(4‐aminophenyl)porphyrin (**Fe**–***p***–**NH**_**2**_–**Cbz**) and iron 5‐(*N*‐benzyloxycarbonyl‐4‐aminophenyl)‐10,15,20‐tris(4‐(trimethylammonio)phenyl)porphyrin (**Fe**–***p***–**TMA**–**Cbz**) for visible‐light‐driven CO_2_ reduction to CO and CH_4_ are reported. Under blue light (447 nm) irradiation, **Fe**–***p***–**NH**_**2**_–**Cbz** and **Fe**–***p***–**TMA**–**Cbz** achieve turnover numbers (TONs) of 20 and 23 for CO, and 6 and 10 for CH_4_, respectively, using a commercially available organic photosensitizer (**Phenox**), triethylamine (TEA) as sacrificial electron donor and trifluoroethanol (TFE) as proton source. In this reaction conditions, **Fe**–***p***–**NH**_**2**_–**Cbz** and **Fe**–***p***–**TMA**–**Cbz** demonstrate catalytic activity comparable to its symmetric counterpart iron 5,10,15,20‐tetra(4‐(trimethylammonio)phenyl)porphyrin (**Fe**–***p***–**TMA**), previously reported by Prof. Marc Robert's group, achieving a TON of 23 for CO and of 11 for CH_4_. Isotopic labeling studies using ^13^CO_2_ confirm that CH_4_ and CO products come from photocatalytic CO_2_ reduction. The results highlight the potential of iron porphyrins as tunable molecular catalysts for photocatalytic CO_2_ reduction beyond two electrons for artificial photosynthesis applications.

## Graphical abstract

Two novel asymmetric porphyrin‐based iron complexes capable of performing photocatalytic CO_2_ reduction (CO_2_R) to CO and CH_4_ with an organic photosensitizer are presented. ^13^CO_2_ labeling studies confirm CH_4_ is formed by CO_2_R. These earth‐abundant based complexes show significant potential for efficient CO_2_R beyond two electrons, joining a single previously reported system as the only molecular catalysts for CH_4_ production via CO_2_R.

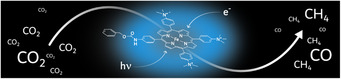

## 1 Introduction

The increasing global reliance on fossil fuels has led to an unprecedented rise in atmospheric CO_2_ levels, significantly contributing to climate change.^[^ ^1^, ^2^, ^3^ ^]^ As such, developing efficient CO_2_ conversion technologies is an urgent scientific and technological goal. In this regard, a promising strategy involves using solar energy either to generate electricity for electrochemical CO_2_ reduction (CO_2_R) or to directly drive its transformation into fuels and chemicals.^[^ ^4^, ^5^, ^6^, ^7^, ^8^, ^9^ ^]^ Several heterogeneous and homogeneous systems have been developed in the last decades for electro‐ and photocatalytic CO_2_R.^[^ ^4^, ^10^, ^11^, ^12^ ^]^ Heterogeneous catalysts can produce different products ranging from CO to CH_4_ and even multicarbon compounds, albeit with low selectivity.^[^ ^13^ ^]^ On the other hand, homogeneous catalysts typically produce two‐electron reduction products like CO and formate.^[^ ^14^ ^]^ Despite this limitation, molecular systems have shown remarkable selectivity for CO_2_R and enabled detailed mechanistic studies, which are crucial for developing more efficient and more selective catalysts.^[^ ^4^, ^10^, ^12^, ^15^, ^16^, ^17^, ^18^, ^19^, ^20^, ^21^, ^22^, ^23^, ^24^, ^25^, ^26^, ^27^, ^28^, ^29^, ^30^, ^31^, ^32^, ^33^ ^]^

Among molecular systems, recent studies on Fe‐porphyrins have demonstrated their ability for electrochemical CO_2_R to CO, with selectivity modulated by ligand modifications, as reported by Costentin, Robert, Savéant and coworkers.^[^ ^34^, ^35^ ^]^ Moreover, Robert's group demonstrated that a tetraphenyl Fe porphyrin bearing trimethylammonio substituents at the *para*‐positions of the four phenyl rings (**Fe**–***p***–**TMA**) was able to perform CO_2_R to CH_4_ under photocatalytic conditions using visible light irradiation, in combination with iridium photosensitizers or phenoxazine derivative organic dyes.^[^ ^36^, ^37^, ^38^ ^]^ These systems achieved up to 17% selectivity for CH_4_ production from CO_2_ together with CO and H_2_ depending on the photocatalytic conditions. To the best of our knowledge, this is the only example of a transition metal‐based molecular complex for photocatalytic CO_2_R to CH_4_, underscoring the reaction's difficulty. Other examples of molecular transition metal‐based systems for CO_2_R to CH_4_ operate under electrocatalytic conditions (see SI Table S3–S4, Supporting Information).^[^ ^33^, ^39^, ^40^ ^]^

Therefore, further exploration of **Fe**–***p***–**TMA** and derivative systems is interesting to better understand the role of the positive charges of the *p*‐TMA ligand in the CO_2_R selectivity. Furthermore, the effect of eliminating one of the four trimethylammonio groups on the selectivity for photocatalytic CO_2_R is unknown. In this context, we propose that replacing one of the positive trimethylammonio groups with a derivatizable protected amino functional group can allow for exploring bifunctional effects or even adding anchoring capacity to the molecular system via the protected amino group. These anchoring capabilities would allow their heterogenization onto different supports upon covalent attachment, from electrodes to soft materials, to increase their stability and selectivity, and even to work in aqueous solutions.^[^ ^5^, ^12^, ^41^ ^]^

**Figure 1:**
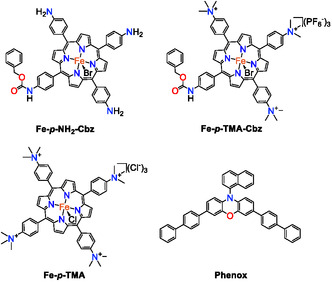
Structures of the novel molecular CO_2_R asymmetric Fe porphyrin complexes developed in this work **Fe−** ***p*** **−NH**_**2**_ **−Cbz** and **Fe−** ***p*** **−TMA−Cbz**, and the previously reported **Fe–** ***p*** **–TMA**, together with the organic photosensitizer **Phenox** used in this work.

Herein, we report the synthesis and photocatalytic activity of two novel asymmetric iron porphyrin complexes (**Figure** **1**) based on *meso*‐tetraaminophenylporphyrin: iron 5‐(*N*‐benzyloxycarbonyl‐4‐aminophenyl)‐10,15,20‐tris(4‐aminophenyl)porphyrin (**Fe**–***p***–**NH**_**2**_–**Cbz**) where one of the amino groups is functionalized with a benzyl carbamate, and iron 5‐(*N*‐benzyloxycarbonyl‐4‐amino phenyl)‐10,15,20‐tris(4‐(trimethylammonio)phenyl) porphyrin (**Fe**–***p***–**TMA**–**Cbz**) where the three remaining amino groups are fully quaternarized to trimethylammonio groups. In combination with the commercially available organic photosensitizer 3,7‐di([1,1'‐biphenyl]‐4‐yl)‐10‐(naphthalen‐1‐yl)‐4a,10a‐dihydro‐10H‐phenoxazine (**Phenox**) under blue light irradiation (447 nm), these complexes show remarkable photocatalytic activity for visible‐light‐driven CO_2_R to CO and CH_4_. Together with the previously reported iron tetra(*p*‐N,N,N‐trimethylanilinium)porphyrin (**Fe**–***p***–**TMA**), these are rare examples of first‐row molecular transition metal complexes capable of reducing CO_2_R to CH_4_ under homogenous photocatalytic conditions reported to date.

## 2 Results and Discussion

### 2.1 Synthesis of Ligands and Complexes

**Scheme 1:**
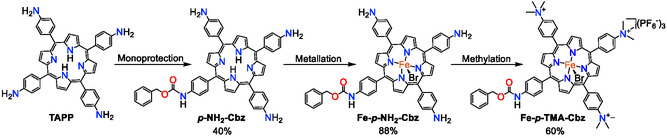
Synthesis of the asymmetric **Fe‐** ***p*** **‐NH**_**2**_ **‐Cbz** and **Fe‐** ***p*** **‐TMA‐Cbz** iron porphyrin complexes.

We have developed a new method to synthesize two new asymmetric iron(III) porphyrin complexes derived from the tetraphenylporphyrin scaffold. We started with the monoprotection of the commercially available 5,10,15,20‐tetra(*p*‐aminophenyl)porphyrin ligand (TAPP) with the Cbz group, due to its stability and facile removal by Pd/C hydrogenation, by adapting a previously reported procedure (**Scheme** **1**).^[^ ^42^ ^]^ The Cbz group serves as a proxy for other peptide bond‐linked modifications onto the ligand.^[^ ^42^, ^43^ ^]^ The presence of four equivalent amino positions in the porphyrin necessitates kinetic control of the reaction to prevent the formation of a product mixture. Cooling and diluting the reaction mixture enabled the isolation of the monoprotected product (*p*–NH_2_–Cbz) in 40% yield (see SI Figure S1, Supporting Information, and Section 2). The undesired multiprotected products can be deprotected to recover the starting TAPP material and recycled.

The iron complex was formed upon *p*–NH_2_–Cbz ligand metalation with excess Fe(II) bromide in anhydrous tetrahydrofuran (THF), adapted from a previously reported procedure,^[^ ^44^ ^]^ forming **Fe**–***p***–**NH**_**2**_–**Cbz** in 88% yield. A subsequent methylation reaction was carried out in a one‐pot two‐step fashion, using MeI and MeOTf as methylating agents, to render **Fe**–***p***–**TMAP**–**Cbz**. To overcome the limitations of nuclear magnetic resonance (NMR) analysis due to the paramagnetism of iron complexes, their zinc analogs were synthesized. However, the Zn complexes’ limited solubility hindered the obtention of high‐quality spectra. Demetallation of the final complex to obtain the free ligands allowed for full NMR characterization of the novel ligand, *p*‐TMA‐Cbz. The ^1^H‐NMR spectra feature the appearance of a (‐N(CH_3_)_3_)^+^ peak at 4.17 ppm and a strong downshift of the *ortho‐* and *meta‐*protons of the three trimethylphenylammonio groups, while the signals corresponding to the Cbz protecting group and its phenyl group are preserved (see SI Figure S18 to S22, Supporting Information for details).

### 2.2 Electrochemical Characterization

The two new complexes were characterized by cyclic voltammetry (CV) in N,N‐dimethylformamide (DMF) solutions with 0.1 M H_2_O and under N_2_ and CO_2_ atmosphere. First, the CVs of **Fe**–***p***–**NH**_**2**_–Cbz and **Fe**–***p** **–*** TMA–**Cbz** under N_2_ atmosphere show two pseudoreversible waves: the first at −0.90 and −0.79 V *vs* Standard Hydrogen Electrode (SHE), for **Fe**–***p***–**NH**_**2**_–**Cbz** and **Fe**–***p***–**TMA**–**Cbz**, respectively, associated to a Fe^II/I^ process, and the second at −1.53 and −1.34 V *vs* SHE, for **Fe**–***p***–**NH**_**2**_–**Cbz** and **Fe**–***p***–**TMA**–**Cbz**, respectively, associated to a Fe^I/0^ process (see Figure S23, Supporting Information). For the new complexes, the redox potential to access the catalytically active Fe^0^ species is slightly more cathodically shifted than the previously reported **Fe**–***p***–**TMA** catalyst (−1.26 V *vs* SHE in DMF).^[^ ^36^ ^]^ Under CO_2_, the electrochemical wave associated with the Fe^II/I^ redox process for **Fe**–***p***–**NH**_**2**_–Cbz is shifted to a more cathodic potential, suggesting an interaction between the complex and CO_2_, which will be further studied in the future. In contrast, under CO_2_, the electrochemical waves associated with the Fe^I/0^ redox process shift to slightly more positive values for both complexes, indicating a fast reaction between the electrochemically generated Fe^0^ species and CO_2_ on the time scale of the CV experiment, as previously observed.^[^ ^26^ ^]^

### 2.3 Photocatalytic Studies

Complexes **Fe**–***p***–**NH**_**2**_–**Cbz** and **Fe**–***p***–**TMA**–**Cbz** were studied for photocatalytic CO_2_R in anhydrous DMF using **Phenox** as photosensitizer, TFE as a source of protons, and TEA as a sacrificial electron donor, in a Parallel light‐emitting diode (LED) Photoreactor from Treellum Technologies with an irradiation wavelength of *λ* = 447 nm.^[^ ^45^, ^46^, ^47^, ^48^ ^]^ An aliquot of the reaction headspace was subjected to chromatographic analysis using a gas chromatography coupled to a thermal conductivity detector and a flame ionization detector (GC‐TCD/FID) to quantify the reaction products (see SI for details). The activity of **Fe**–***p***–**TMA** was also studied under these conditions, similar to the ones used by Robert and coworkers,^[^ ^36^, ^38^ ^]^ but with our irradiation setup.

As shown in **Table** **1** (entries 1–3), all studied complexes showed photocatalytic activity for CO_2_R, producing CO and CH_4_ in all cases, obtaining a turnover number (TON) CH_4_ of 6, 10, and 11 (8%, 22% and 32% selectivity with a quantum yield of 0.0003%, 0.0005%, and 0.0005%, respectively) and a TON CO of 20, 23, and 23 (27%, 52% and 68% selectivity) for complexes **Fe**–***p***–**NH**_**2**_–**Cbz**, **Fe**–***p***–**TMA**–**Cbz,** and **Fe**–***p***–**TMA**, respectively. No H_2_ was detected as a byproduct for **Fe**–***p***–**TMA** under our experimental conditions, while the new asymmetric Fe porphyrin complexes showed a TON H_2_ of 47 and 12, respectively, being the tricationic complex **Fe**–***p***–**TMA**–**Cbz** more selective for CO_2_R. The new tricationic complex **Fe**–***p***–**TMA**–**Cbz** exhibited similar activity for CH_4_ production as the previously reported tetracationic **Fe**–***p***–**TMA** complex in our conditions, albeit with lower selectivity (22% vs 32% selectivity, Table 1 entries 2 and 3). However, the obtained TON for CH_4_ and CO was lower than the previously reported for the **Fe**–***p***–**TMA** complex (140 TON CO and 29 TON CH_4_), and no H_2_ was detected.^[^ ^38^ ^]^ The differences in the observed reactivity can be attributed to different experimental and light irradiation setups, notably the use of a solar simulator with AM1.5G and IR/UV filtering in the former setup^[^ ^38^ ^]^ versus a parallel blue LED photoreactor (*λ* = 447 nm, ≈1 W per LED) with precise temperature control in our system, as well as different equipment for the analysis of products (see SI for details).^[^ ^3^, ^45^, ^46^, ^47^, ^48^, ^49^ ^]^

**Table 1:** Photocatalytic CO_2_R to CH_4_ by complexes **Fe**‐***p***‐**NH**_**2**_‐**Cbz**, **Fe**‐***p***‐**TMA**‐**Cbz**, and **Fe**‐***p***‐**TMA**, using **Phenox** as photosensitizer under blue light irradiation (447 nm). Reported TON after 96 h.

Entry	Catalyst	TON (Selectivity)		
		H_2_	CO	CH_4_
1	**Fe**‐***p***‐**NH**_**2**_‐**Cbz**	47 ± 5 (65%)	20 ± 4 (27%)	6 ± 1 (8%)
2	**Fe**‐***p***‐**TMA**‐**Cbz**	12 ± 1 (26%)	23 ± 4 (52%)	10 ± 3 (22%)
3	**Fe**‐***p***‐**TMA**	n.d.	23 ± 1 (68%)	11 ± 2 (32%)

General photocatalytic conditions: CO_2_R catalyst (0.01 mM), Phenox (1 mM), in DMF and TFE (0.1 M), using TEA (0.1 M) as sacrificial electron donor, reaction volume 3 mL, under visible light irradiation (447 nm), 25 °C, and CO_2_ atmosphere.

**Figure 2:**
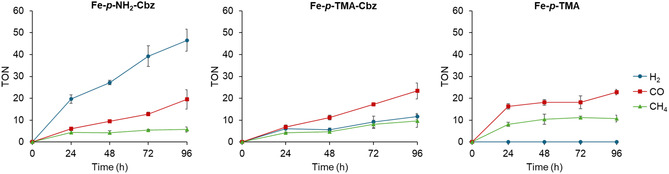
Time‐traces of the photochemical CO_2_R with **Fe‐** ***p*** **‐NH**_**2**_ **‐Cbz**, **Fe‐** ***p*** **‐TMA‐Cbz,** and **Fe‐** ***p*** **‐TMA**. Photocatalytic conditions: CO_2_R catalyst (0.01 mM), **Phenox** (1 mM), in DMF and TFE (0.1 M), TEA (0.1 M), reaction volume 3 mL, *λ* = 447 nm at 25 °C and under CO_2_ atmosphere.

**Figure 3:**
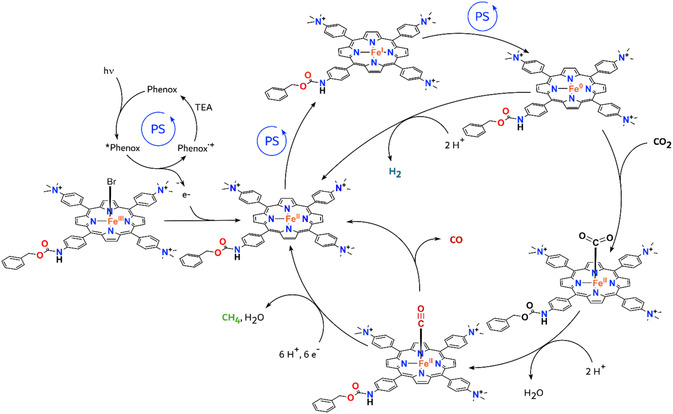
Proposed mechanism for the CO_2_R to CO and CH_4_ by complexes **Fe‐** ***p*** **‐NH**_**2**_ **‐Cbz** and **Fe‐** ***p*** **‐TMA‐Cbz** with **Phenox** as photosensitizer, together with H_2_ production, according to previously reported studies for **Fe‐** ***p*** **‐TMA**.^[^ ^38^ ^]^

As shown in **Figure** **2**, CO is produced upon blue light irradiation (*λ* = 447 nm) at the beginning of the reaction, and CH_4_ production starts after some CO has built up, suggesting that CO is an intermediate in the methane formation process, as previously reported.^[^ ^36^, ^38^ ^]^ Increasing the irradiation time up to 96 h increased the amount of CO_2_R products generated (Figure 2). Control experiments confirmed that the reaction does not proceed in the absence of light irradiation, the CO_2_R catalyst, the photosensitizer, or a CO_2_ atmosphere (Table S1, Supporting Information, entries 1–4). However, control experiments without TEA (electron donor) produced substochiometric CO, CH_4_, or H_2_ versus **Phenox,** with both catalysts, **Fe**–***p***–**NH**_**2**_–**Cbz** or **Fe**–***p***–**TMA**–**Cbz** (Table S1, Supporting Information, entries 6, 7, 9, and 10). This is in line with an oxidative quenching mechanism of the excited state of **Phenox** by the Fe(III) complex to generate the catalytically active species (see the mechanistic discussion below and **Figure** **3**).^[^ ^38^, ^50^ ^]^ Without an electron donor, the oxidized species of the photosensitizer (Phenox^·^^+^) cannot be reduced to regenerate the starting **Phenox**, so the concentration of photosensitizer (1 mM) limits the quantity of products that can be formed until it is all consumed. In all cases, since a 1 mM concentration of **Phenox** in a 3 mL reaction volume can produce 3 μmol of electrons, the number of electrons consumed to form the detected products is equal or below the limit of the total number of electrons that the initial **Phenox** can give. In addition, for the reactions in the absence of TFE (proton source), lower amounts of H_2_ or no H_2_ were detected due to the lower concentration of protons (see Table S1, Supporting Information, entries 5 and 8).

To demonstrate the CH_4_ and CO formation from photocatalytic CO_2_R, we performed ^13^CO_2_ isotopic labeling studies, monitoring the evolved gaseous products on the reaction headspace using an online mass spectrometer (see SI Section 1.6, Figure S24, Supporting Information, for further details). In isotopic labeling experiments conducted under ^13^CO_2_, mass spectrometry analysis identified ^13^CO (*m*/*z* = 29) and ^13^CH_4_ (*m*/*z* = 17) as evolved reaction products. In contrast, photocatalytic experiments performed under nonlabeled CO_2_ atmosphere revealed the formation of ^12^CO (*m*/*z* = 28) and ^12^CH_4_ (*m*/*z* = 16), confirming that both methane and CO originate from photocatalytic CO_2_R.

Considering the previously studied **Fe**–***p***–**TMA** system and its similarity with the herein reported catalysts, we propose a similar CO_2_R photocatalytic mechanism for complexes **Fe**–***p***–**NH**_**2**_–**Cbz** and **Fe**–***p***–**TMA**–**Cbz** using **Phenox** as photosensitizer (Figure 3).^[^ ^38^, ^50^ ^]^ First, upon light absorption, the excited state of **Phenox** (*Phenox) is generated, which undergoes oxidative quenching by the Fe(III) porphyrin to yield the oxidized species Phenox^·^^+^ and the Fe(II) complex that enters the catalytic cycle. Subsequent electron transfer steps generate the catalytically active Fe(0) species. This mechanism aligns with previous studies, where the redox potential of the singlet and triplet excited state of **Phenox** (*Phenox, −1.6 and −1.5 V *vs* SHE, respectively), combined with emission quenching and Stern–Volmer analyses, demonstrated that both excited states are reductive enough to generate the catalytically active Fe(0) species of the **Fe**–***p***–**TMA** porphyrin (−1.26 V *vs* SHE in DMF).^[^ ^36^, ^50^ ^]^ Importantly, these studies showed that the electron donor did not quench the emission of *Phenox, which confirms that the electron transfer proceeds from *Phenox to the Fe catalyst via oxidative quenching, generating Phenox^·^^+^.^[^ ^50^ ^]^ As such, the initial Fe(III) complex undergoes three subsequent one‐electron reductions from *Phenox to yield the catalytically active Fe(0) species.^[^ ^38^, ^50^ ^]^ In our case, with the same organic photosensitizer (**Phenox**), we can consider the same mechanism, since the redox potential of *Phenox can also reduce the complexes of this study to the catalytically active Fe(0) species (E^0^(Fe^I/0^) = −1.53 and −1.34 V *ves* SHE for complexes **Fe**–***p***–**NH**_**2**_–**Cbz** and **Fe**–***p***–**TMA**–**Cbz**, respectively). Then the Fe(0) species can react with protons to yield H_2_ as a byproduct, or with CO_2_ to form a Fe^II^CO_2_ intermediate.^[^ ^38^, ^51^ ^]^ After further protonation and elimination of water, the Fe^II^CO intermediate is generated, which has been detected in a previous study.^[^ ^52^ ^]^ Finally, the Fe^II^CO intermediate can evolve CO as a product, or be further reduced and protonated to finally evolve CH_4_ and restore the initial Fe(II) complex.

## 3 Conclusion

This work presents two novel asymmetric iron porphyrin complexes, **Fe**–***p***–**NH**_**2**_–**Cbz** and **Fe**–***p***–**TMA**–**Cbz,** for photocatalytic CO_2_R to CO and CH_4_ when combined with **Phenox** as photosensitizer. Complexes **Fe**–***p***–**NH**_**2**_–**Cbz** and **Fe**–***p***–**TMA**–**Cbz** yielded TON CH_4_ of 6 and 10 and a TON CO of 20 and 23, respectively, under a CO_2_ atmosphere after 96 h of blue‐light irradiation. These systems showed moderate selectivity for CH_4_ production, comparable to the previously reported **Fe**–***p***–**TMA**. These results demonstrate the huge potential of iron porphyrin systems for the challenging CO_2_R beyond two electrons under photocatalytic conditions. The new reported complexes illustrate that only three positive charges surrounding the catalytic site are enough to produce CH_4_ photocatalytically in moderate selectivity, which opens the possibility to further explore the effect of the charge of the porphyrin ligand in the CO_2_R‐to‐CH_4_ activity and selectivity. Moreover, the protected amino group of these complexes can be further deprotected to explore those systems as heterogenized CO_2_R catalysts under photo‐, electro‐ of photoelectrocatalytic conditions upon their covalent attachment to electrodes or other supports.

Therefore, this work will pave the way for further development of active and selective photo‐ or (photo)electrocatalytic systems for CO_2_R‐to‐CH_4_ and their potential coupling with oxidation reactions as a source of electrons toward sustainable solar fuel production using compartmentalized systems.

## Conflict of Interest

The authors declare no conflict of interest.

## Supporting information

Supplementary Material

## Data Availability

The data that support the findings of this study are available in the supplementary material of this article.
