# Supplementary material for: Novel Asymmetric Iron Porphyrins for Photocatalytic CO2 Reduction to CH4
Source: ChemSusChem. 2025 Jul 17;18(18):e202500715. doi: 10.1002/cssc.202500715 (PMC12456383; doi:10.1002/cssc.202500715)
Supplement: Supplementary file 1 — Supplementary Material [file CSSC-18-e202500715-s001.pdf]

Supplementary Information

Novel Asymmetric Iron Porphyrins for Photocatalytic CO_2 Reduction to CH_4

Edelman J. Espinoza-Suárez,[1,#] Akhmet Bekaliyev,[1,#] Aranza Vital-Grappin, Laura Velasco-Garcia,[2] Laia Subirats Valls,[1] Carla Casadevall[1,2,*]

Affiliations:

[1]Rovira i Virgili University (URV), Carrer de Marcel·lí Domingo, 1, 43007, Tarragona (Spain). [2]Institute of Chemical Research of Catalonia (ICIQ), The Barcelona Institute of Science and Technology, Avinguda dels Països Catalans, 16, 43007, Tarragona (Spain). *Correspondence to: carla.casadevall@urv.cat

[#]These authors contributed equally.

Table of contents

1. General procedures 3
   1.1. Materials 3
   1.2. Physical Methods 3
   1.3. Electrochemistry 3
   1.4. High Resolution Mass Spectrometry (HRMS) 3
   1.5. Elemental analysis 4
   1.6. General procedure for the photocatalytic experiments 4
   1.7. General procedure for the photocatalytic $^{13}CO_2$ isotopic labelling studies 4
2. Synthesis of ligands and complexes and characterization 5
   2.1. Synthesis of *p*-NH_2-Cbz 5
   2.2. Synthesis of iron(III) monoprotected ligand complex (Fe-*p*-NH_2-Cbz) 9
   2.3. Synthesis of Fe-*p*-TMA-Cbz 10
   2.4. Synthesis of zinc monoprotected ligand complex (Zn-*p*-NH_2-Cbz) 12
   2.5. Synthesis of Zn-*p*-TMA-Cbz 14
   2.6. Demetallation to render *p*-TMA-Cbz 16
3. Electrochemical characterization 20
4. Photocatalytic studies 21
5. $^{13}CO_2$ isotopic labelling studies 21
6. Determination of the Quantum Yield 22
7. Reported catalysts for CO_2R to CH_4 23
8. References 25

1. General procedures

1.1. Materials

Reagents and solvents were acquired from commercial suppliers and used without further purification, except where specified. The following substances were obtained from Sigma Aldrich: iodomethane (MeI), methyl trifluoromethanesulfonate (MeOTf), 2,6-lutidine, iron (II) bromide, palladium on activated carbon, 10% Pd, ammonium hexafuorophosphate, 1-naphthalene-10-phenoxazine (phenox), sodium hydroxide, hexane, bathocuproine, xantphos, 5,10,15,20-tetrakis(4-trimethyl-ammoniophenyl)porphyrin tetra(p-toluene sulfonate), triethylamine (TEA), acetone, ethtyl acetate (EtOAc), anhydrous acetonitrile (CH_3CN anh.), anhydrous N,N-dimethylformamide (DMF anh.), tetrahydrofuran (THF) methanol, tetrakis(acetonitrile)copper(I) hexafluorophosphate, diethyl ether, deuterated acetone, deuterated methanol, and deuterated chloroform. Hydrochloric acid 37% and 1,4-dioxane anhydrous were bought from Carlo Erba. Iron(III) 5,10,15,20-(tetraphenyl)porphyrin chloride, 5-(4-aminophenyl)-10,15,20-(triphenyl)porphyrin, and 5-(4-aminophenyl)-10,15,20-(tri-4-sulfonatophenyl)porphyrin triammonium were bought from Porphychem. Acetic acid (HOAc) 10% was bought from ChemLab, sodium sulfate anhydrous was bought from PanReac AppliChem, and 5,10,15,20-tetrakis(4-aminophenyl)porphyrin was bought from Tokyo Chemical industry. All solvents underwent thorough degassing and were stored under anaerobic conditions.

1.2. Physical Methods

NMR spectra were recorded on Bruker Avance Neo 400 HMz, Agilent NMR System 400 MHz and AVIII500 spectrometers. All ^{1}H chemical shifts are reported in ppm and have been internally calibrated to the residual protons of the deuterated solvent. The ^{13}C chemical shifts have been internally calibrated to the carbon atoms of the deuterated solvent. The coupling constants were measured in hertz (Hz).

UV-Vis spectra were recorded on an Agilent UV-Vis flexible Cary 3500 spectrophotometer employing 1 cm quartz cells at 300 K.

Attenuated total reflection infrared (ATR- IR) spectra were recorded on a Fourier Transform Infrared (FTIR) Bruker spectrometer model Alpha with an ATR accessory (spectral range from 4000 to 400 cm^{-1}).

1.3. Electrochemistry

Electrochemical experiments were carried out using a SP-150 BioLogic® potentiostat. Cyclic voltammetry (CV) measurements were carried out under nitrogen or carbon dioxide atmospheres by using 1 mM solutions of the metal complexes in a DMF solution of 100 mM H_2O and 100 mM tetrabutylammonium hexafluorophosphate ($TBAPF_6$), unless otherwise indicated. A single-compartment three-electrode cell was employed, with a 3 mm glassy carbon working electrode, Pt wire counter electrode and a saturated calomel electrode (SCE) as reference. All the potentials are reported *vs* saturated calomel electrode (SCE). CVs were recorded at scan rate of 100 mV/s. The working electrodes were polished by using 0.05 µM alumina powder (CHInstruments) on a polishing pad wet with distilled water, followed by rinsing with distilled water/acetone and air dried.

1.4. High Resolution Mass Spectrometry (HRMS)

Samples ionized by electrospray ionization (ESI) were introduced as a 0.2 ppm MeOH solution in a MicroTOF Focus II (Bruker) instrument, set in positive ion mode (m/z range: 50-3000, capillary: 4500 V, end plate offset: -500 V, nebulizer pressure 0.4 bar, dry heater temperature 180°C, dry gas flow 4 l/min).

1.5. Elemental analysis

Samples were analyzed by combustion in oxygen atmosphere at 1200°C using an CHNS Thermo Scientific Flash 2000 elemental analyzer, and quantification of the combustion products via GC. Sulfanilamide (PN33825100 from ThermoFischer Scientific) was used as reference substance.

1.6. General procedure for the photocatalytic experiments

A parallel blue LED (λ = (447 ± 20) nm, 1030 mW @ 700 mA) photoreactor from Treellum Technologies®[1-4] with 25 equivalent positions was used to perform the photocatalytic reactions, allowing for strict reaction temperature control by a high precision thermoregulation Hubber K6 cryostat. The photoreactor is typically powered with a power supply of 75 V and a current of 0.7 A (2.1 W per LED).

All catalytic reactions were conducted in a 10 mL septum-capped vial under vigorous stirring using an orbital stirrer and irradiating at 447 nm for 96h under carbon dioxide atmosphere at 25 ºC, unless otherwise indicated. Catalytic assays were performed using Phenoxazine as photosensitizer (1 mM) and the Fe porphyrin as CO_2 reduction catalyst (0.01 mM) in dimethylformamide (DMF) and 0.1 M 2,2,2-trifluoroethanol (TFE) as solvent mixture, together with 0.1 M of triethanolamine (TEA) as sacrificial electron donor, unless specified otherwise. After reaction completion, an aliquot of the reaction headspace was subjected to gas chromatographic analysis. The headspace products CO, CH_4 and H_2, were quantified by gas chromatography with an Agilent 7820A GC System equipped with columns Washed Molecular Sieve 5A, 2m x 1/8'' OD, Mesh 60/80 SS and Porapak Q, 4m x 1/8'' OD, SS. Mesh: 80/100 SS, coupled to a Thermal Conductivity Detector (TCD) and a Flame Ionization Detector (FID). Helium was used as carrier gas. Manual injections were made using a 100 μL graduated sample-lock gastight syringe (Hamilton). The quantification of CO, H_2 and CH_4 obtained was measured through the interpolation of a previous calibration using different syngas or methane mixtures in a purged CO_2 atmosphere.

1.7. General procedure for the photocatalytic $^{13}CO_2$ isotopic labelling studies

Fe-*p*-TMA-Cbz (0.091 mg, 6x10^{-5} mmol, final concentration 0.01 mM), **Phenox** (3.7 mg, 6x10^{-3} mmol, final concentration 1 mM), TFE (0.043 mL, 0.6 mmol, final concentration 0.1 M), and TEA (0.084 mL, 0.6 mmol, final concentration 0.1 M) were dissolved in anhydrous DMF (4.67 mL) in a 10 mL crimped vial that was purged with unlabelled $^{12}CO_2$ or with labelled $^{13}CO_2$, prior visible light irradiation with a single LED photoreactor (λ = 447 nm) at room temperature. In all cases, the reaction headspace of the vial was connected to an atmospheric pressure quadrupole mass spectrometer (Pfeiffer Omnistar GSD 301 C1; m/z 16, 17, 28, 29, 44, 45 were monitored) to monitor the derived gaseous reaction products.

Since the methane production is slow for this catalyst, in order to accumulate enough product in the reaction headspace, we designed a system to be able to connect the reaction headspace to the mass spectrometer with an intermediate valve to allow monitoring the reaction headspace before and after irradiation, allowing enough quantity of CO_2R derived products to accumulate. First, the reaction vial was connected to the mass spectrometer and the headspace sampled to monitor the target ions at time 0 before irradiation, then the valve was closed and the reaction left stirring under blue light (447 nm) irradiation for 72 h. Finally, after the reaction, the valve was opened to allow sampling of the headspace. The ion counting in the headspace before light irradiation was subtracted to the one after light irradiation to have the neat production of CO and CH_4 (and ^{13}CO and $^{13}CH_4$) during the reaction in the non-labelled and labelled experiments. For all cases, first a blank reaction was performed by omitting the catalyst and photosensitizer to test the system before doing the experiments under $^{12}CO_2$ or $^{13}CO_2$ atmosphere.

2. Synthesis of ligands and complexes and characterization

Fe-*p*-TMA was synthesized following a reported procedure.[5]

The synthesis of the new asymmetric neutral and tricationic porphyrin ligands and their corresponding iron and zinc complexes followed the scheme shown in figure S1. Details for each step are covered in the following sections.

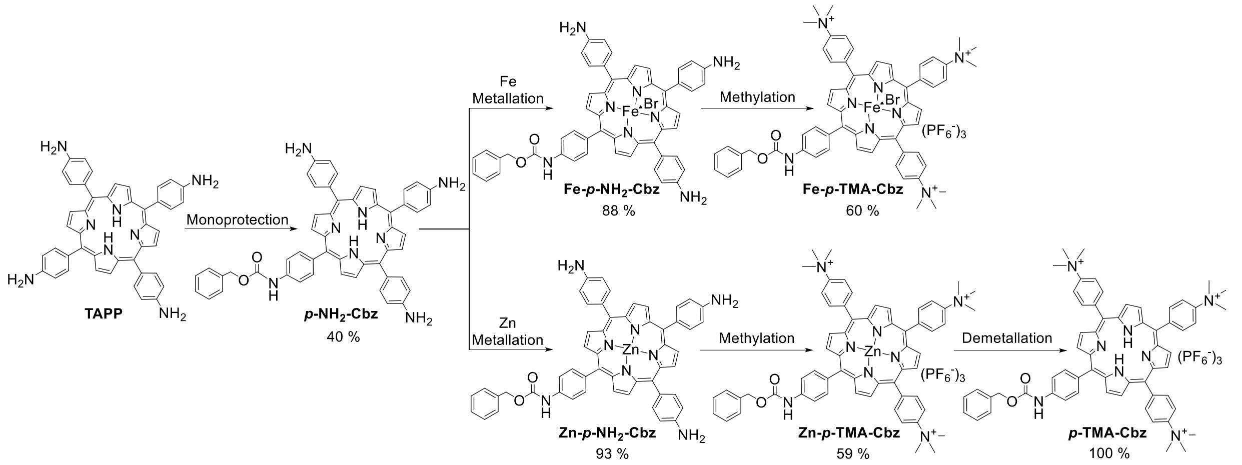

Figure S1. General synthetic scheme.

2.1. Synthesis of *p*-NH2-Cbz

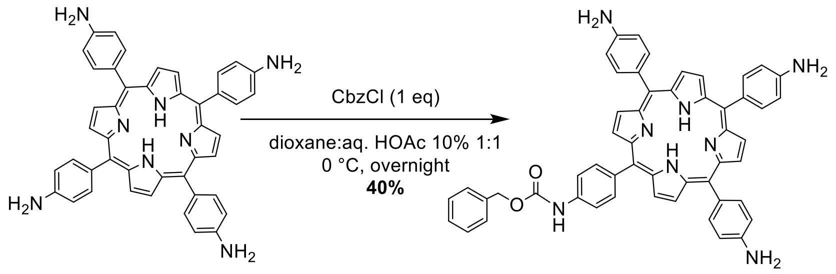

201.2 mg (0.298 mmol) of 5,10,15,20-tetrakis(4-aminophenyl)porphyrin was dissolved in 40 mL of 10% aqueous acetic acid and cooled in an ice bath. In another vessel, 40 µL (0.3 mmol; 1 eq) of benzyl chloroformate chloride (CbzCl) were diluted in 40 mL of 1,4-dioxane. The CbzCl solution was added dropwise under vigorous stirring to the porphyrin solution over 30 minutes. The reaction mixture was left to stir and warm up to room temperature overnight. The mixture was made basic and the porphyrin products extracted in diethyl ether. The resulting organic phase was adsorbed onto silica and separated via flash chromatography using EtOAc:hexane 70:30 as the starting mobile phase, increasing to 100 % EtOAc. The desired product was obtained as a dark purple solid. (96.8 mg, 0.120 mmol, 40 % yield) after drying in vacuo.

^{1}H NMR (401 MHz, Acetone-d_6) δ 9.20 (s, 1H), 8.96 (m, 8H), 8.17 (d, *J* = 8.5 Hz, 2H), 8.04 (d, *J* = 8.5 Hz, 2H), 7.92 (d, *J* = 8.3 Hz, 6H), 7.54 (d, *J* = 8.1 Hz, 2H), 7.45 (t, *J* = 7.2 Hz, 1H), 7.39 (d, *J* = 7.2 Hz, 1H), 7.10 (d, *J* = 8.4 Hz, 6H), 5.32 (s, 2H), -2.59 (s, 2H).

^{13}C NMR (126 MHz, Acetone-d_6) δ 154.63 (NH**C**OOR), 149.27 (C-NH_3), 137.97 (meso C), 136.36 (**B** C), 135.73 (α C), 131.18 (pyrrolic CH), 131.12 (pyrrolic CH), 129.38 (m-Cbz), 129.05 (o-Cbz), 128.94 (p-Cbz), 121.76 (p-NH2), 118.78 (p-carbamate), 117.48 (β C), 113.64 (***A*** C), 67.10 (CH_2).

IR (ATR), ν (cm^{-1}): 3311 (carbamate N-H stretch, w), 3022 (N-H stretch, w), 1699 (carbamate C=O stretch, m), 1207 (Ar-N stretch).

UV/Vis (CH_2Cl_2, nm (mol^{-1}dm^{3}cm^{-1})): λ_{max} (ε)= 428 (4.56x10^5), 520 (1.92x10^4), 561 (1.88x10^4), 597 (7.09x10^3), 656 (1.04x10^4).

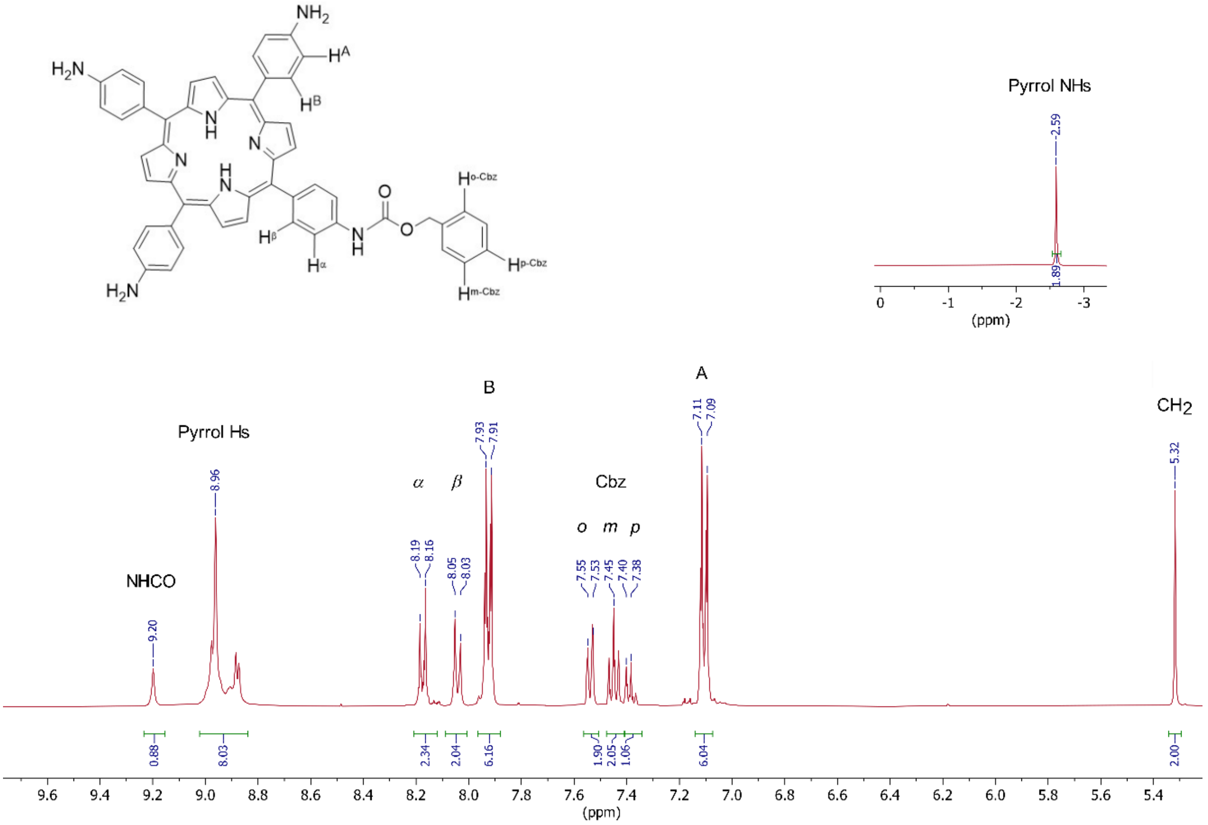

Figure S2. ^{1}H-NMR (500 MHz, 298 K, acetone-d$_6$) spectrum of ***p*-NH$_2$-Cbz**.

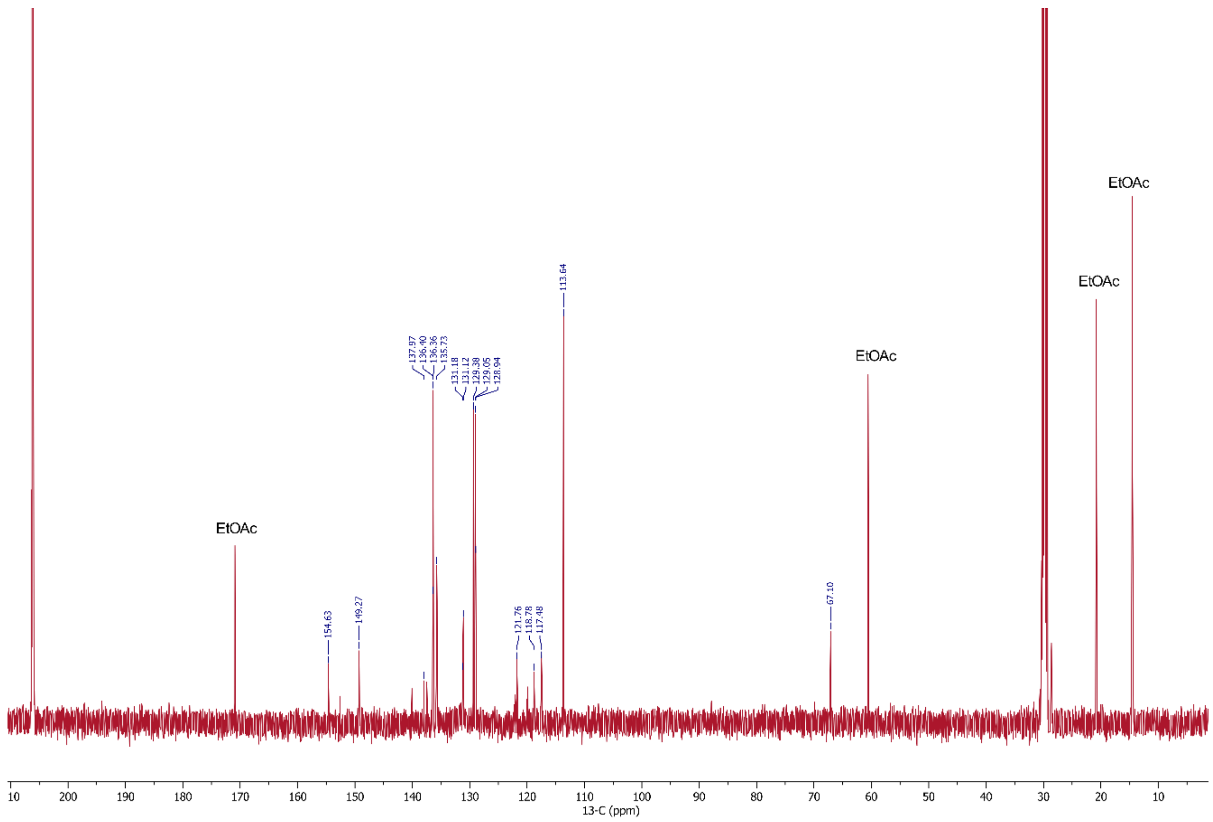

Figure S3. ^{13}C-NMR (126 MHz, 298 K, acetone-d_6) spectrum of ***p*-NH$_2$-Cbz.**

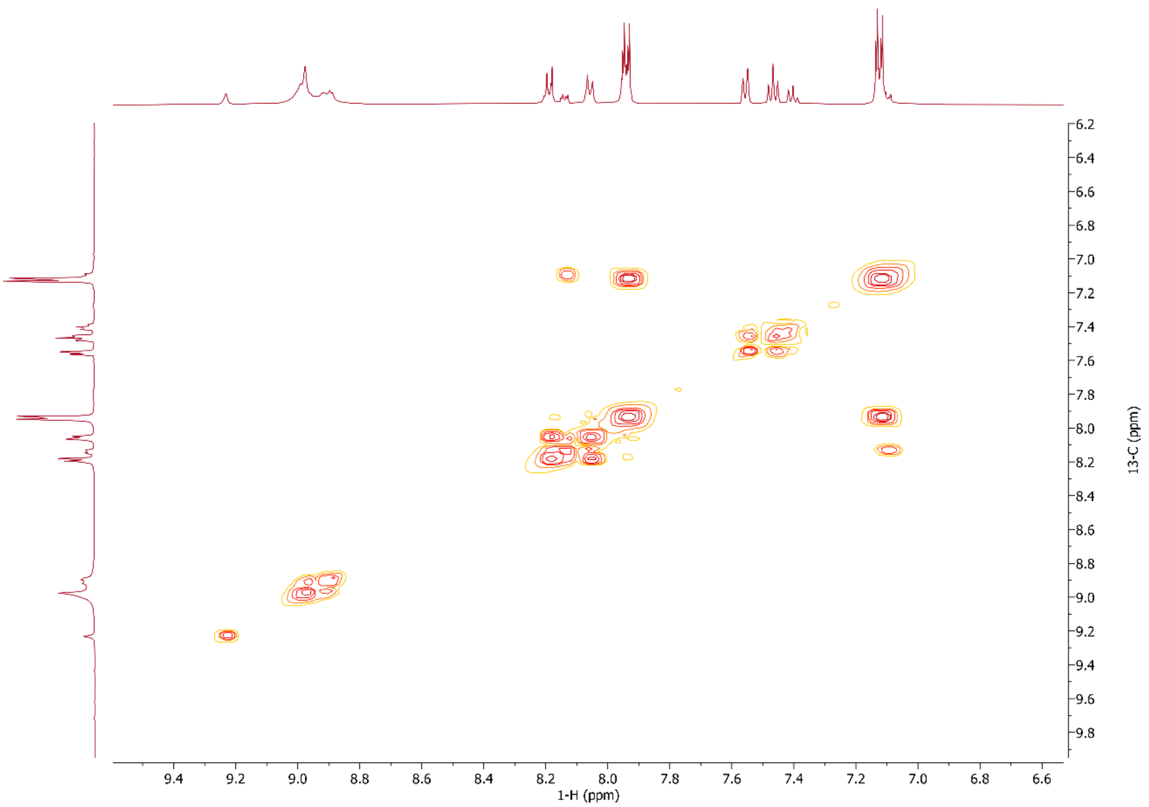

Figure S4. ^{1}H-^{1}H-COSY NMR (500 MHz, 298 K, acetone-d_6) spectrum of ***p*-NH$_2$-Cbz**.

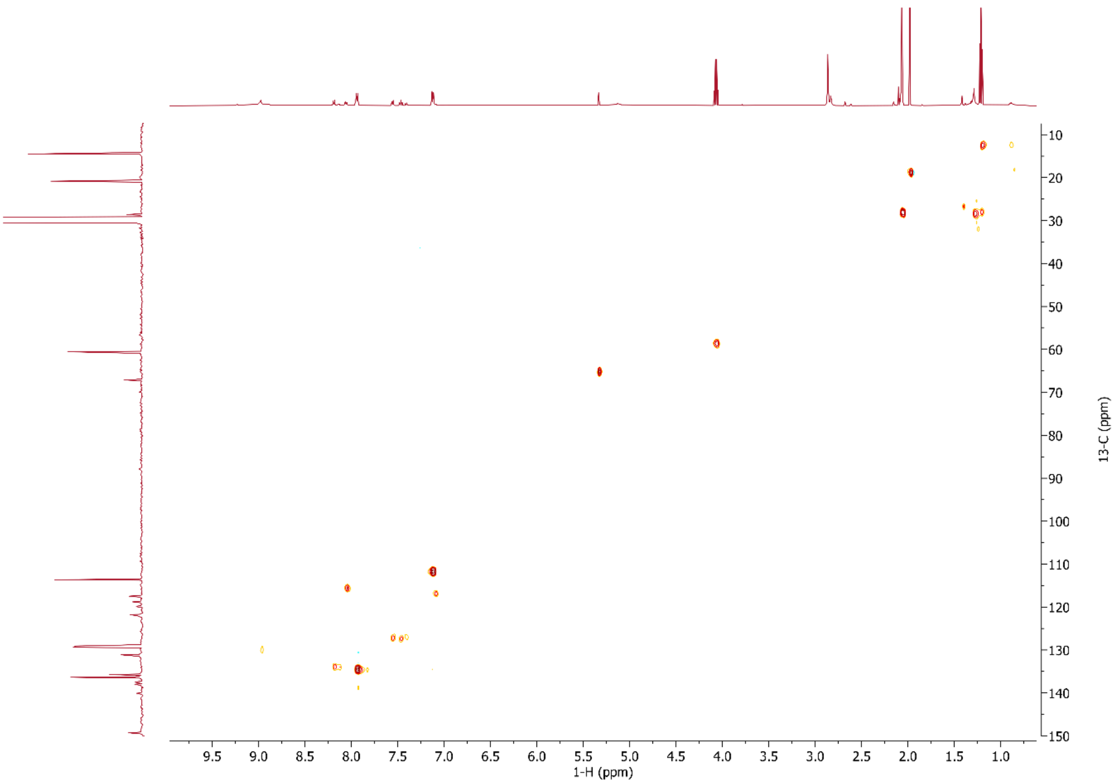

Figure S5. ^{1}H-^{13}C-HSQC NMR (500 MHz, 298 K, acetone-d_6) spectrum of ***p*-NH$_2$-Cbz**.

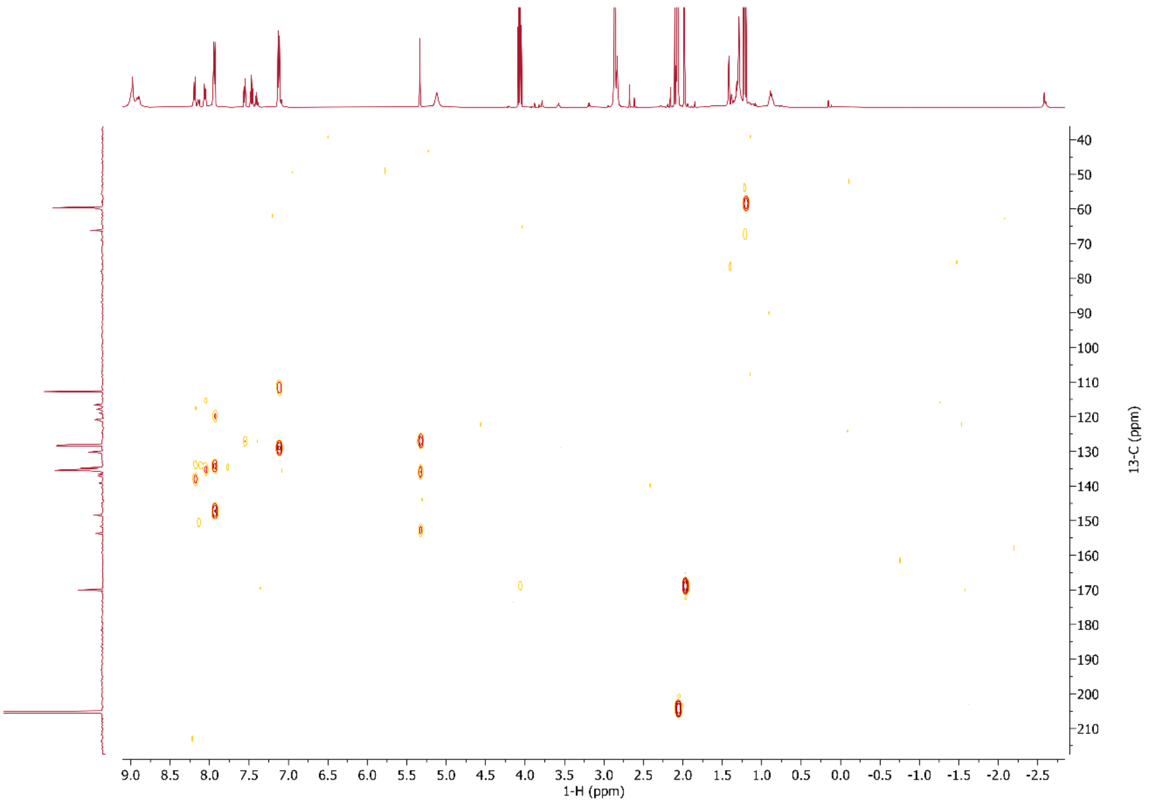

Figure S6. ^{1}H-^{13}C-HMBC NMR (500 MHz, 298 K, acetone-d_6) spectrum of ***p*-NH$_2$-Cbz**.

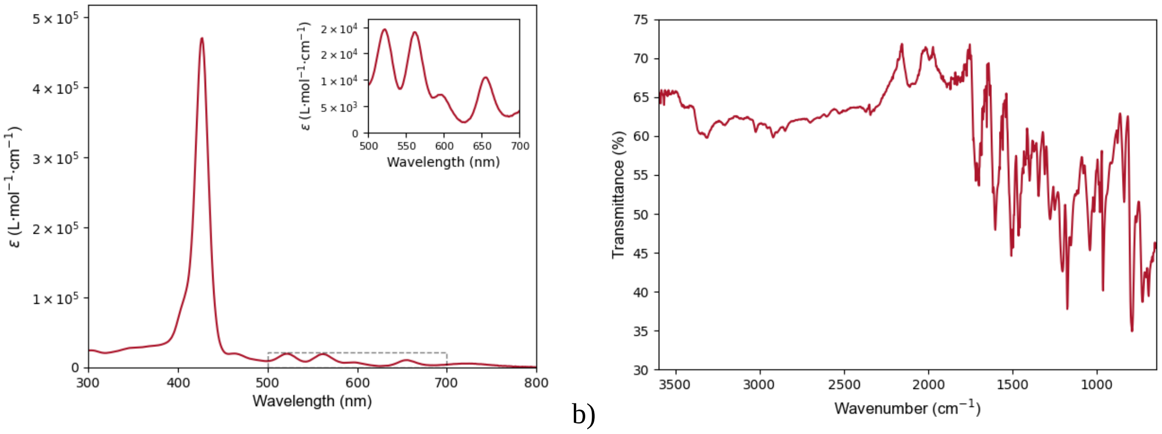

Figure S7. Spectral characterization of ***p*-NH$_2$-Cbz**: a) UV-Vis spectrum, b) IR-ATR spectrum.

2.2. Synthesis of iron(III) monoprotected ligand complex (Fe-*p*-NH$_2$-Cbz)

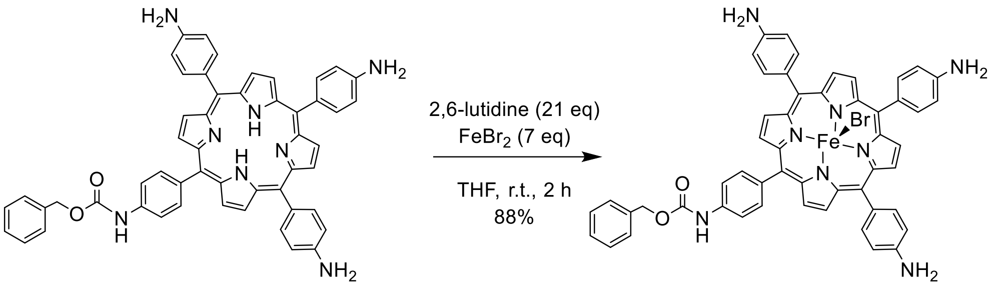

99.8 mg (123 µmol) of ***p*-NH$_2$-Cbz** were dissolved in 16 mL of dry THF, to which 300 µL of 2,6-lutidine were added (2.59 mmol, 21 eq). To this, a solution of 191.6 mg of $FeBr_2$ (0.88 mmol, 7.1 eq) dissolved in 16 mL of dry THF was added and the resulting mixture was stirred for 2 h at room temperature under argon atmosphere. The solvent was removed and the residue redissolved in CH_2Cl_2 and washed with water until the water phase was colorless to remove excess iron salt. The organic phase was then dried over Na_2SO_4, filtered, and dried in vacuo. **Fe- *p*-NH$_2$-Cbz** was obtained as a dark green powder (103.1 mg; 109.4 µmol, 88% yield).

IR (ATR), ν (cm^{-1}): 3328 (carbamate N-H stretch, w), 2920 (N-H stretch, w), 1604 (carbamate C=O stretch, m), 995 (Fe-dependent in-plane porphyrin deformation[6]).

UV/Vis (CH_2Cl_2, nm (mol^{-1}dm^{3}cm^{-1})): λ_{max} (ε)= 420 (9.31x10^4), 575 (6.43x10^3), 620 (5.71x10^3).

HRMS: [M-Br] exp. 862.2455, calc. 862.2456.

Elemental analysis calcd (%) for $C_{52}H_{38}N_8O_2FeBr \cdot HBr \cdot (THF)_5$: C 62.48, H 5.75, N 8.09; found: C 63.00, H 6.42, N 7.35.

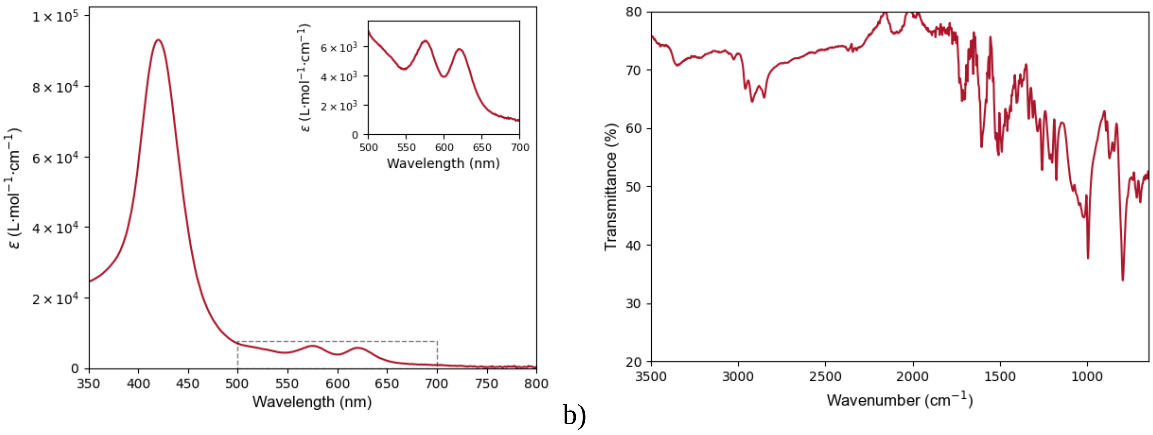

a) b)

Figure S8. Spectral characterization of **Fe-*p*-NH2-Cbz**: a) UV-Vis spectrum, b) IR spectrum.

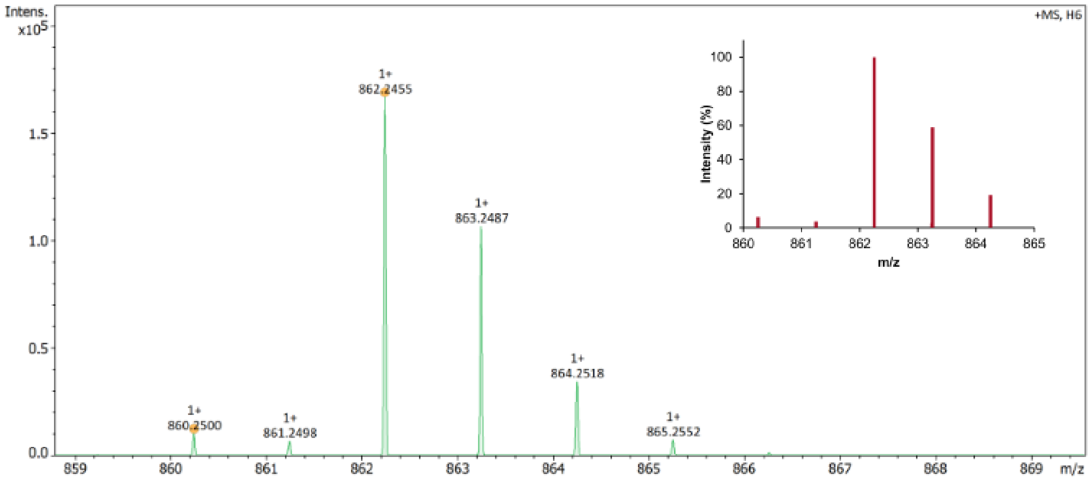

Figure S9. Experimental and simulated (top-right) MALDI-HRMS fragmentation spectra. The main peak corresponds to [M-Br]$^+$.

2.3. Synthesis of Fe-*p*-TMA-Cbz

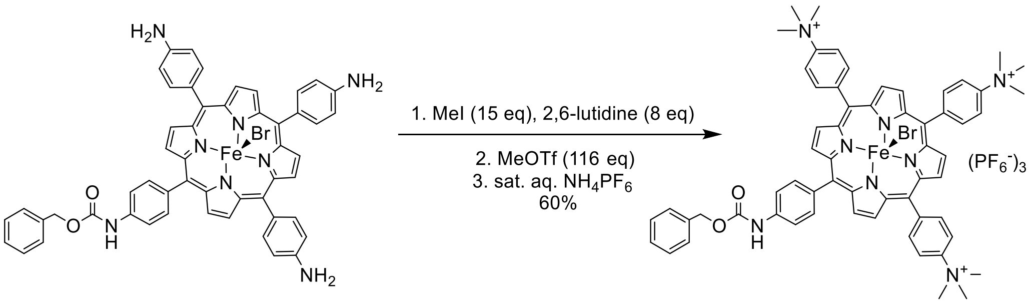

In a vial closed to air, 20 mg of **Fe-*p*-NH2-Cbz** (21.2 µmol) was dissolved in 1.5 mL of anhydrous DMF, resulting in a green solution. To this, 16 µL of 2,6-lutidine (168 µmol, 8 eq) and 20 µL of MeI (321 µmol, 15 eq) were added, quickly changing color to dark red, and the reaction mixture was stirred overnight. The next day, MeOTf (250 µL, 2470 µmol, 116 eq) was added, and the reaction mixture was stirred for an additional 24 hours at 100°C, during which the color gradually shifted to red-orange. The reaction mixture was then quenched with water and precipitation of the porphyrin was performed by

addition of aqueous saturated ammonia hexafluorophosphate. A dark brown precipitate formed, which was washed several times with water and finally with ether and dried in vacuo. **Fe-*p*-TMA-Cbz** was obtained as a dark brown powder (19.2 mg; 12.7 µmol, 60% yield).

IR (ATR), ν (cm^{-1}): 3654 (carbamate N-H stretch, w), 1494 (Ar-N^{+} stretch, m), 1708 (carbamate C=O stretch, m), 961 (Fe-dependent in-plane porphyrin deformation,[6] w).

UV/Vis (CH_3OH, nm (mol^{-1}dm^{3}cm^{-1})): λ_{max} (ε)= 413 (8.52x10^{5}), 569 (7.04x10^{4}), 609 (4.62x10^{4}).

HRMS: [M-3PF_6-Br+MeO]$^{3+}$ exp. 340.8122, calc. 340.8091.

Elemental analysis calcd (%) for $C_{61}H_{59}N_8O_2FeBrP_3F_{18}\cdot(Et_2O)_4$: C 51.28, H 5.53, N 6.21; found: C 51.25, H 5.31, N 5.68.

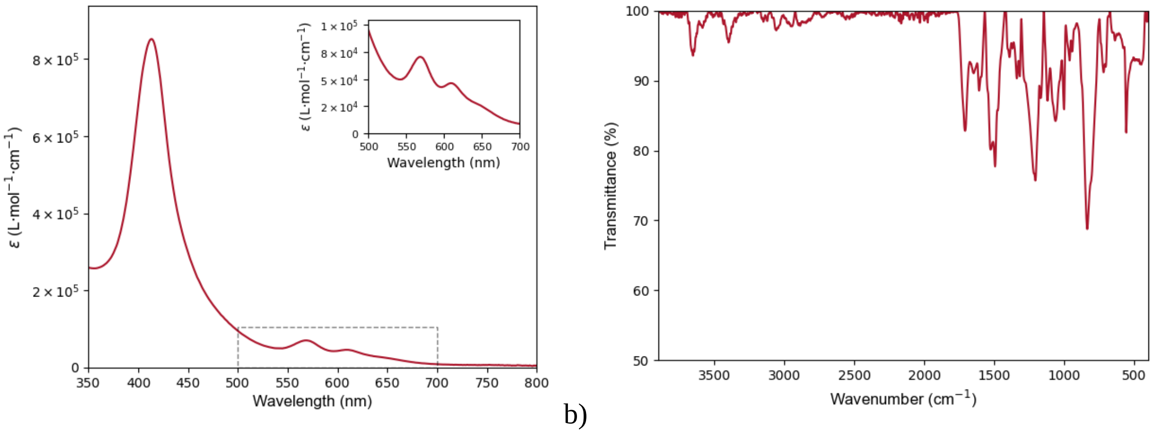

a) b)

Figure S10. Spectral characterization of **Fe-*p*-TMA-Cbz**: a) UV-Vis spectrum, b) IR spectrum.

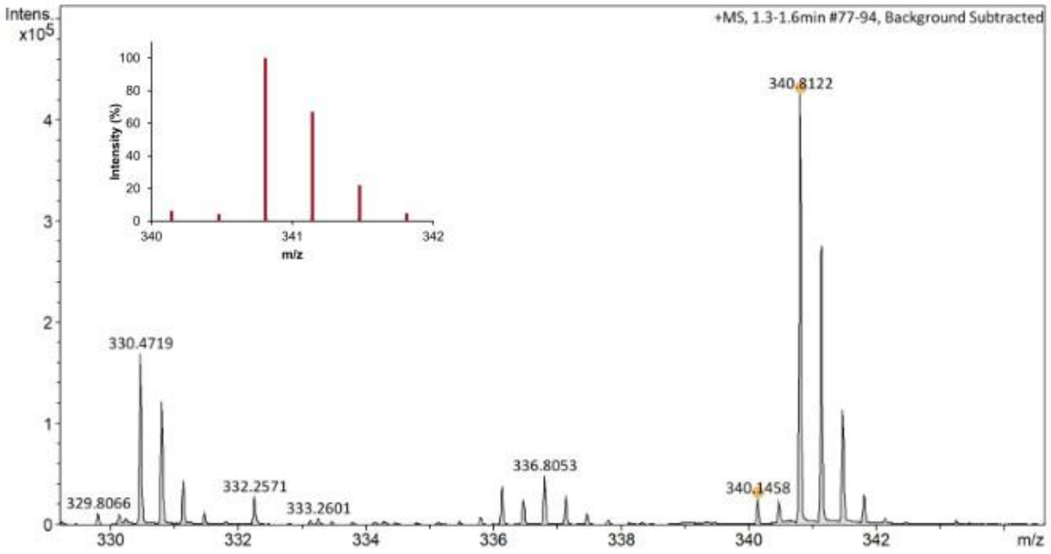

Figure S11. Experimental and simulated (top-left) ESI-HRMS fragmentation spectra. The main peak corresponds to [M-3PF_6-Br+MeO] $^{3+}$.

2.4. Synthesis of zinc monoprotected ligand complex (Zn-*p*-NH_2-Cbz)

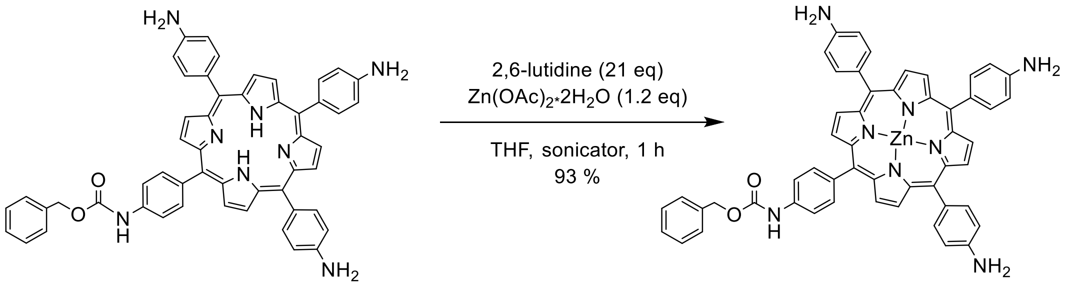

30.2 mg (37 µmol) of ***p*-NH_2-Cbz** were dissolved in 3 mL of dry THF, to which 8.5 µL of 2,6-lutidine were added (73 µmol, 2 eq). To this, a solution of 9.7 mg of Zn(whatsa´p$CH_3COO)_2$·$2H_2O$ (44 µmol, 1.2 eq) dissolved in 3 mL of THF was added and the resulting mixture was sonicated for 1 h. The reaction mixture turned from red to green and a TLC showed no remaining starting material left. The solvent was removed and the residue redissolved in CH_2Cl_2 and washed with water and brine. The organic phase was then dried over Na_2SO_4, filtered, and dried in vacuo. **Zn-*p*-NH_2-Cbz** was obtained as a purple powder (30.3 mg; 35 µmol, 93% yield).

^{1}H NMR (401 MHz, acetone-*d*$_6$) δ 9.17 (s, 1H), 9.00 – 8.77 (m, 8H), 8.13 (t, *J* = 8.3 Hz, 4H), 8.00 (d, *J* = 8.0 Hz, 2H), 7.96 (d, *J* = 7.7 Hz, 2H), 7.89 (d, *J* = 7.8+ Hz, 6H), 7.55 (d, *J* = 7.4 Hz, 2H), 7.46 (t, *J* = 7.3 Hz, 2H), 7.40 (d, *J* = 7.3 Hz, 1H), 7.16 (d, *J* = 7.9 Hz, 2H), 7.04 (d, *J* = 8.0 Hz, 6H), 7.00 – 6.88 (m, 2H), 5.32 (s, 2H). No clear attribution of the phenyl protons can be made from the existing data. Several ionization equilibria species may be present.

^{13}C NMR (126 MHz, acetone-*d*$_6$) δ 135.18, 128.48, 128.14, 112.54, 54.07, 31.74, 22.44, 13.46. No clear attribution of the signals can be made from the existing data.

IR (ATR), ν (cm^{-1}): 3366 (carbamate N-H stretch, w), 2919 (N-H stretch, w), 1715 (carbamate C=O stretch, m), 1177 (Ar-N stretch), 532 (Zn-dependent in-plane porphyrin deformation,[6] m).

UV/Vis (CH_3OH, nm (mol^{-1}dm^{3}cm^{-1})): λ_{max} (ε)= 428 (5.70x10^5), 522 (5.28x10^3), 562 (2.31x10^4), 604 (2.00x10^4).

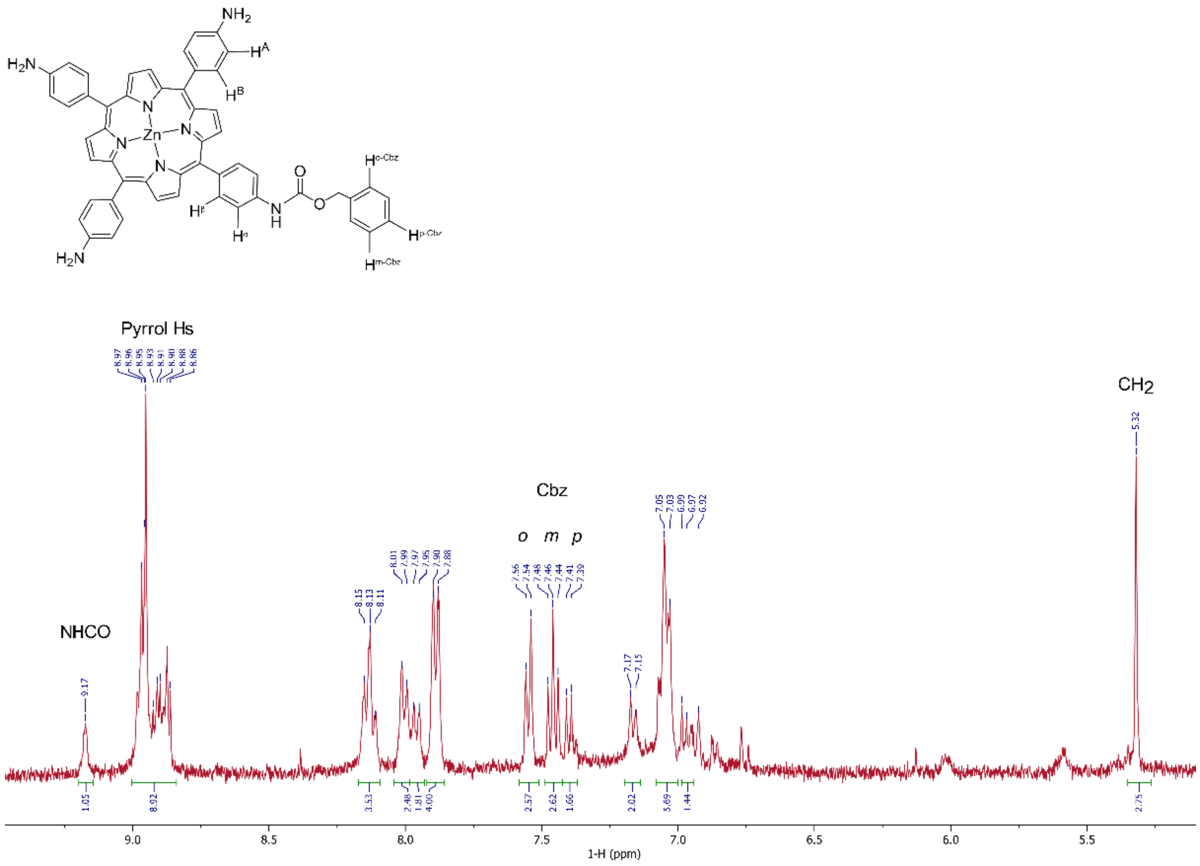

Figure S12. ^{1}H-NMR (500 MHz, 298 K, acetone-d_6) spectrum of **Zn-*p*-NH$_2$-Cbz**.

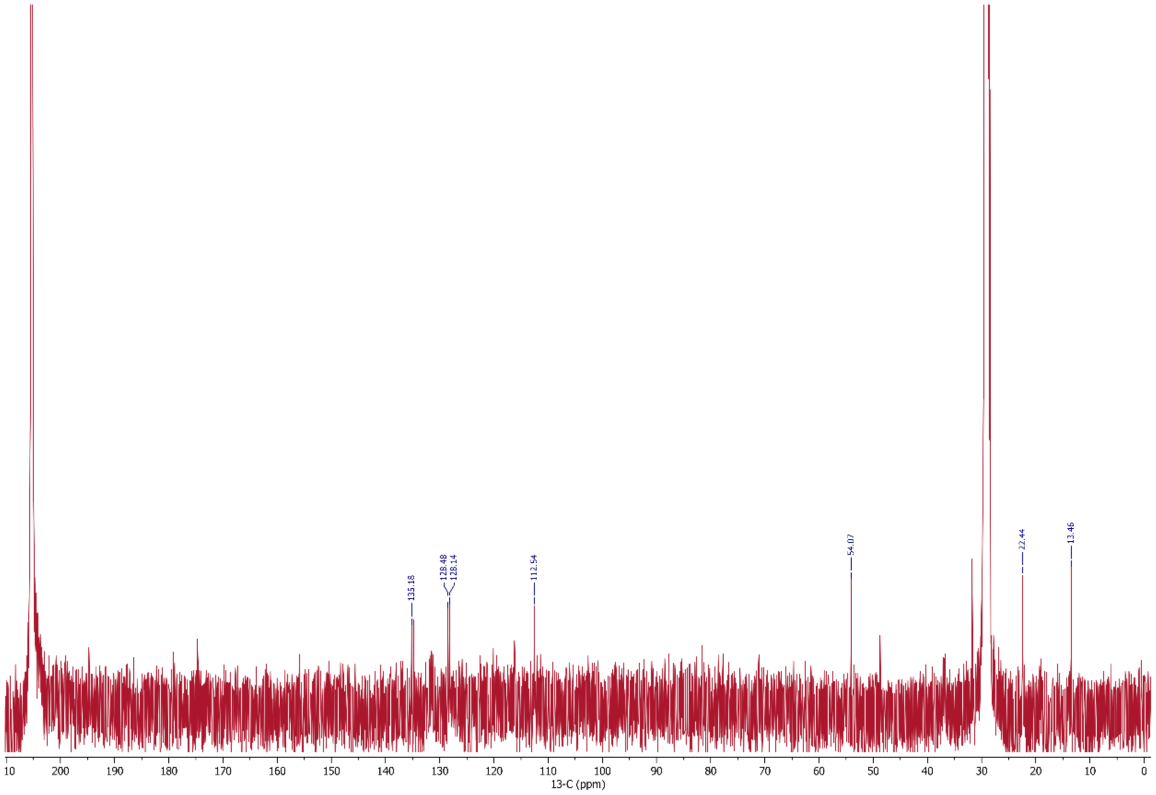

Figure S13. ^{13}C-NMR (500 MHz, 298 K, acetone-d_6) spectrum of **Zn-*p*-NH$_2$-Cbz**.

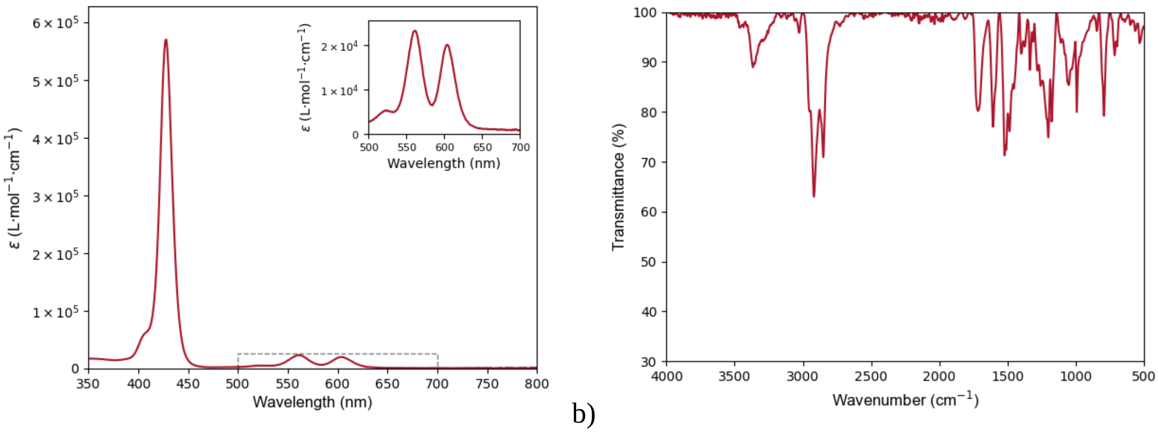

Figure S14. Spectral characterization of **Zn-*p*-NH$_2$-Cbz**: a) UV-Vis Spectrum, b) IR spectrum.

2.5. Synthesis of Zn-*p*-TMA-Cbz

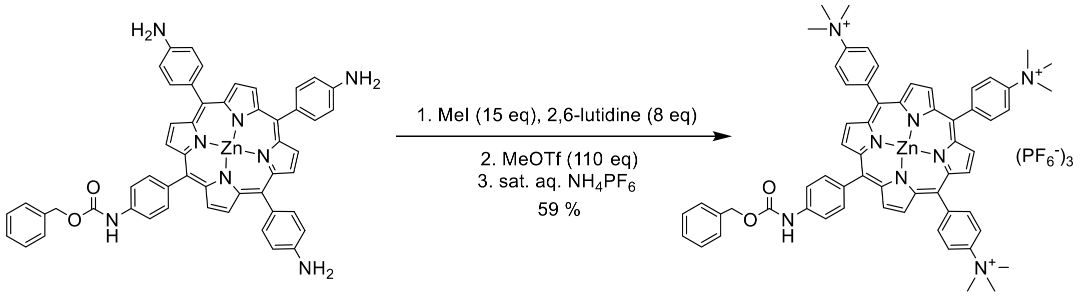

In a vial under Ar atmosphere 23.9 mg (27 µmol) of **Zn-Cbz-*p*-ATPP** were dissolved in 2 mL of anhydrous DMF. To this, 25 µL of 2,6-lutidine (216 µmol, 8 eq) and 25 µL of MeI (401 µmol, 15 eq) were added, rendering a green solution which was stirred overnight at room temperature. Then, 300 µL of MeOTf (2630 µmol, 97 eq) were added and the reaction was held at 100 °C for 24 h. After this time the cationic metalloporphyrin was precipitated using saturated aqueous NH_4PF_6. The precipitate was washed several times with water and finally with ether. The obtained precipitate was dried in vacuo to render 23.1 mg (16.2 µmol, 59% yield) of a brown powder.

^{1}H NMR (500 MHz, acetone-d_6) δ 9.30 (s, 1H), 9.04 – 8.71 (m, 8H), 8.57 – 8.49 (m, 8H), 8.15 (d, J = 8.1 Hz, 2H), 8.04 (d, J = 7.6 Hz, 2H), 7.55 (d, J = 7.5 Hz, 2H), 7.47 (t, J = 7.3 Hz, 2H), 7.43 – 7.37 (m, 1H), 5.33 (s, 2H), 4.21 (d, J = 6.4 Hz, 27H).

^{13}C NMR (126 MHz, acetone-d_6) δ 136.43, 135.65, 129.39, 129.05, 119.45, 58.19, 54.97.

IR (ATR), ν (cm^{-1}): 3638 (carbamate N-H stretch), 3403 (N-H stretch, w), 2925 (C-H stretch, w), 1709 (carbamate C=O stretch, m), 1491 (Ar-N$^+$ stretch, m), 557 (Zn-dependent in-plane porphyrin deformation,[6] m).

UV/Vis (CH_3OH, nm (mol^{-1}dm^{3}cm^{-1})): λ_{max} (ε)= 428 (5.70x10^5), 522 (5.28x10^3), 562 (2.31x10^4), 604 (2.00x10^4).

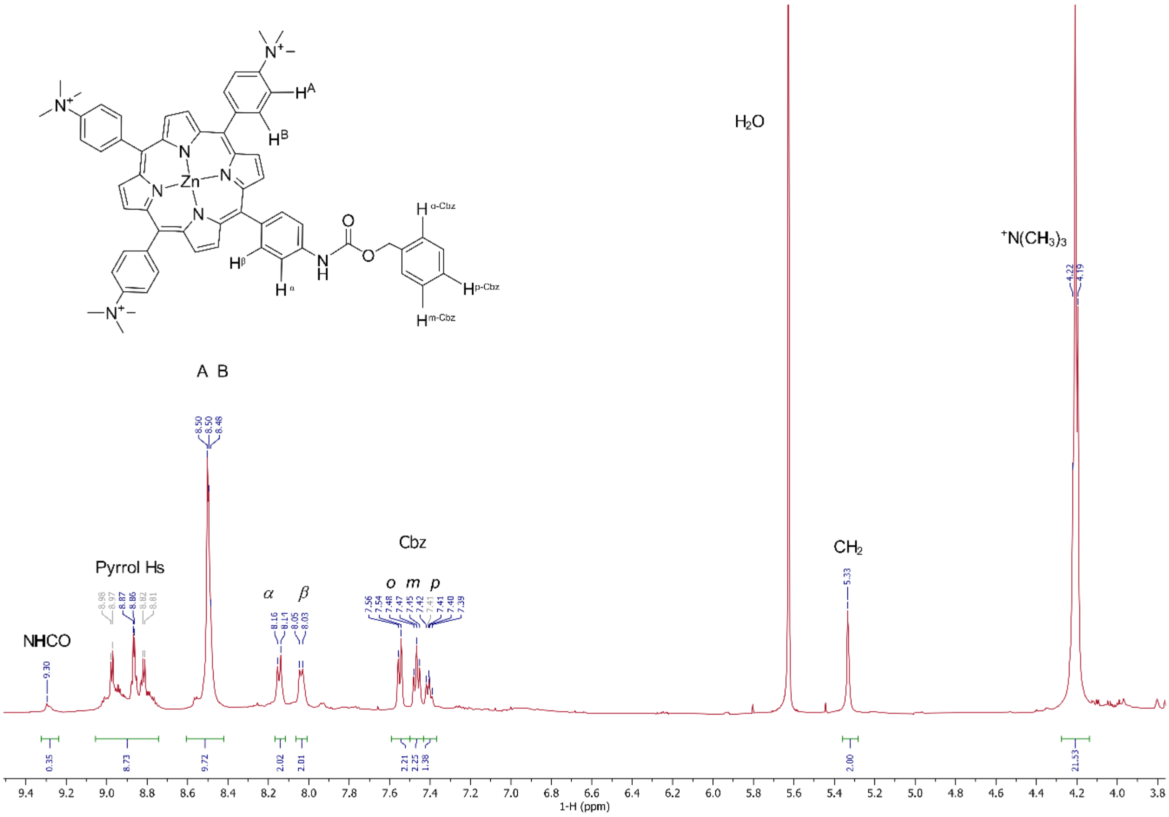

Figure S15. ^{1}H-NMR (500 MHz, 298 K, acetone-d6) spectrum of **Zn-*p*-TMA-Cbz** .

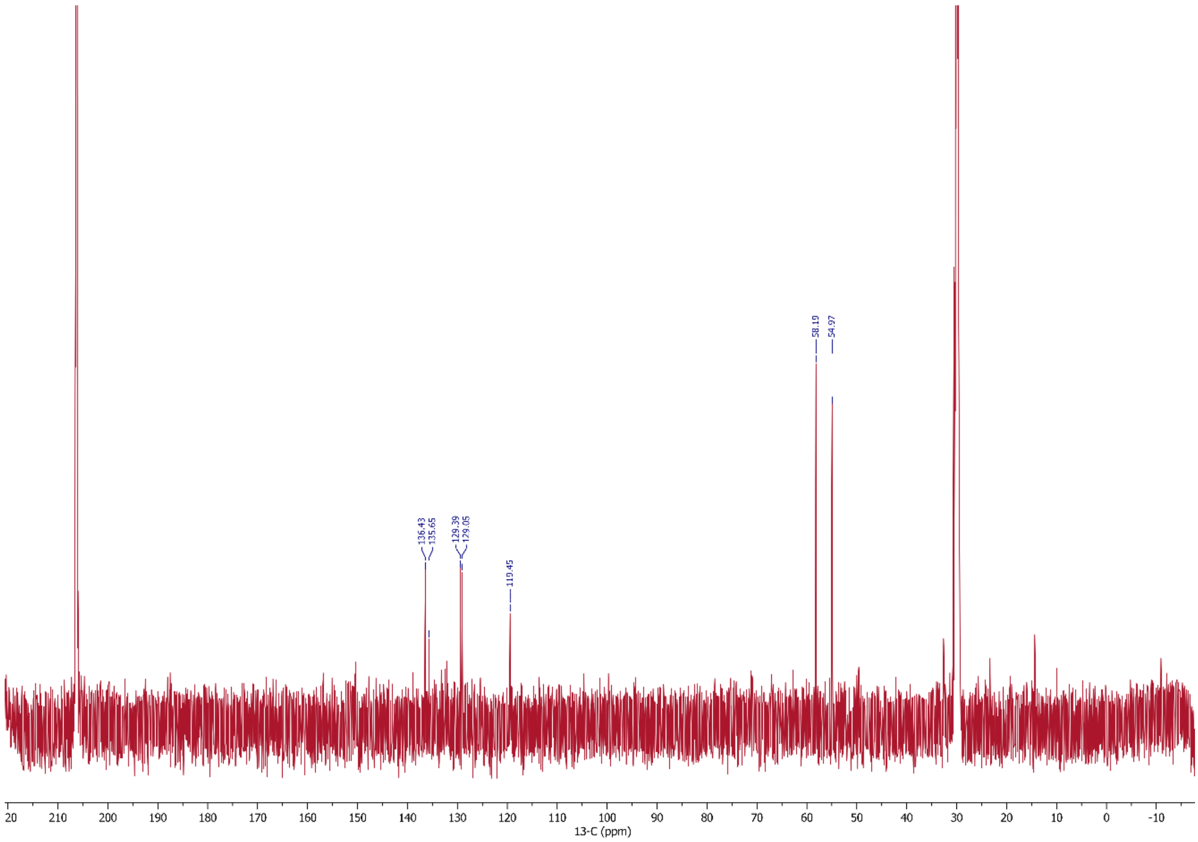

Figure S16. ^{13}C-NMR (126 MHz, 298 K, acetone-d6) spectrum of **Zn-*p*-TMA-Cbz** .

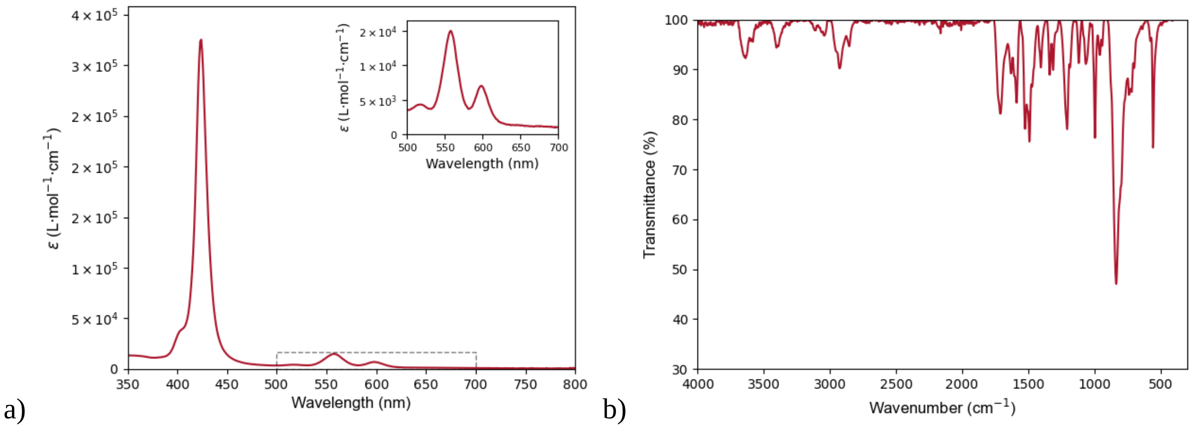

Figure S17. Spectral characterization of **Zn-*p*-TMA-Cbz**: a) UV-Vis Spectrum, b) IR spectrum.

2.6. Demetallation to render *p*-TMA-Cbz

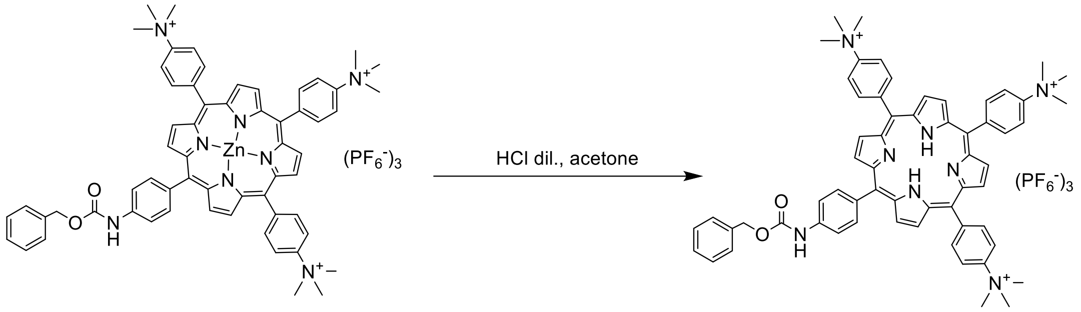

A portion of **Zn-*p*-TMA-Cbz** were dissolved in acetone and poured dropwise into a diluted HCl acetone solution. Immediately, the formation of a green precipitate. The solution was centrifuged, decanted and washed several times with acetone and dried under vacuo to obtain a brown powder with quantitative yield. The washes preserved a green color, and on standing precipitated more of the product **p-TMA-Cbz**. The NMR characterization of the product is shown below, which confirms the quaternization of three amino moieties and the preservation of the Cbz protecting group on the fourth one.

^{1}H NMR (500 MHz, methanol-d_4) δ 9.23 (s, 1H), 8.98 (d, *J* = 4.7 Hz, 2H), 8.87 (d, *J* = 4.6 Hz, 2H), 8.86 (d, *J* = 4.4 Hz, 2H), 8.82 (d, *J* = 4.6 Hz, 2H), 8.47 (s, 12H), 8.15 (d, *J* = 8.1 Hz, 2H), 8.03 (d, *J* = 8.0 Hz, 2H), 7.54 (d, *J* = 7.6 Hz, 2H), 7.46 (t, *J* = 7.4 Hz, 2H), 7.40 (t, *J* = 7.2 Hz, 1H), 5.32 (s, 2H), 4.17 (s, 27H).

^{13}C NMR (126 MHz, methanol-d_4) δ 151.52 (NHCOOR), 150.64 (*p*-$N^+(CH_3)_3$), 150.42 (meso C), 147.77 (C-$N^+(CH_3)_3$), 136.46 (***A*** C), 135.69 (α C), 133.27(pyrrolic CH), 132.48 (pyrrolic CH), 132.22 (pyrrolic CH), 129.41 (*m*-Cbz), 129.06 (*o*-Cbz), 129.00 (*p*-Cbz), 119.46 (***B*** C), 119.27 (*p*-carbamate), 117.27 (β C), 66.11 (CH_2), 58.21 (^+N-CH_3).

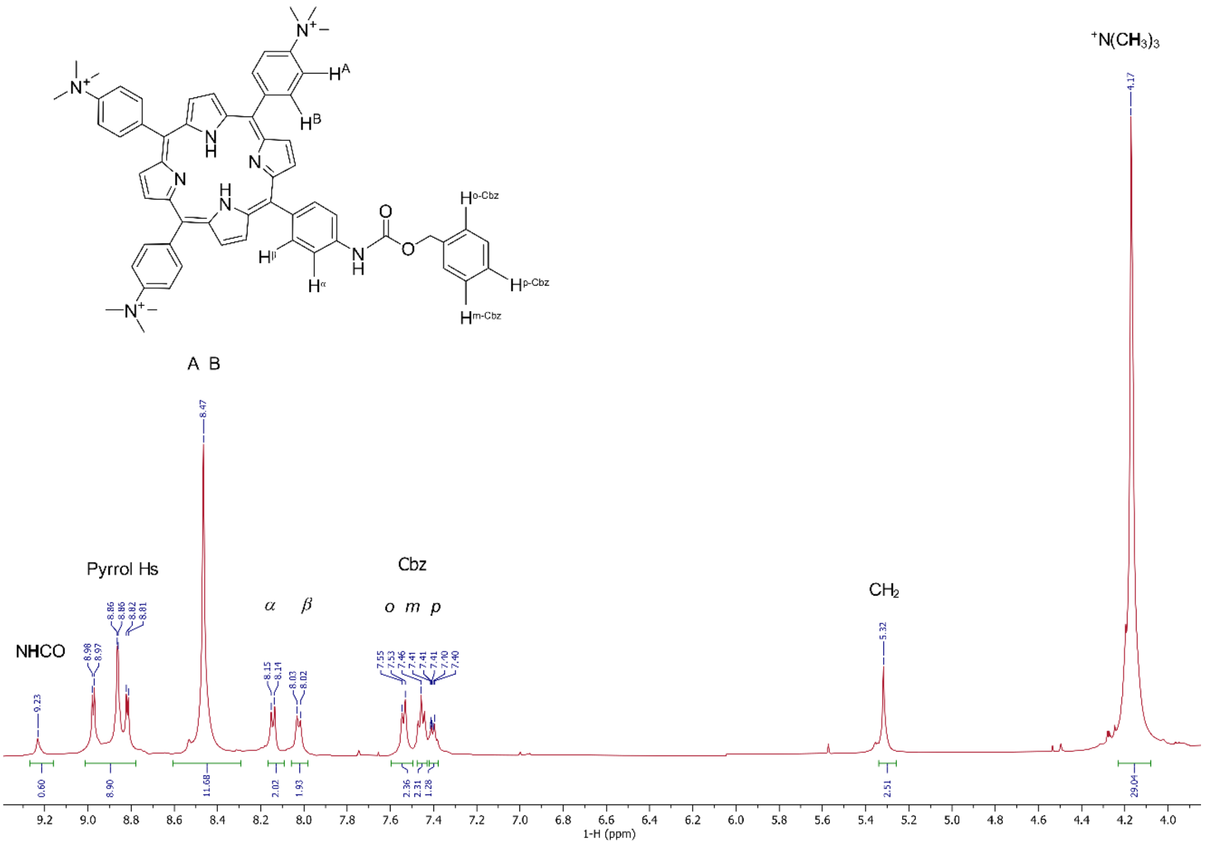

Figure S18. ^{1}H- NMR (500 MHz, 298 K, methanol-d_4) spectrum of ***p*-TMA-Cbz**.

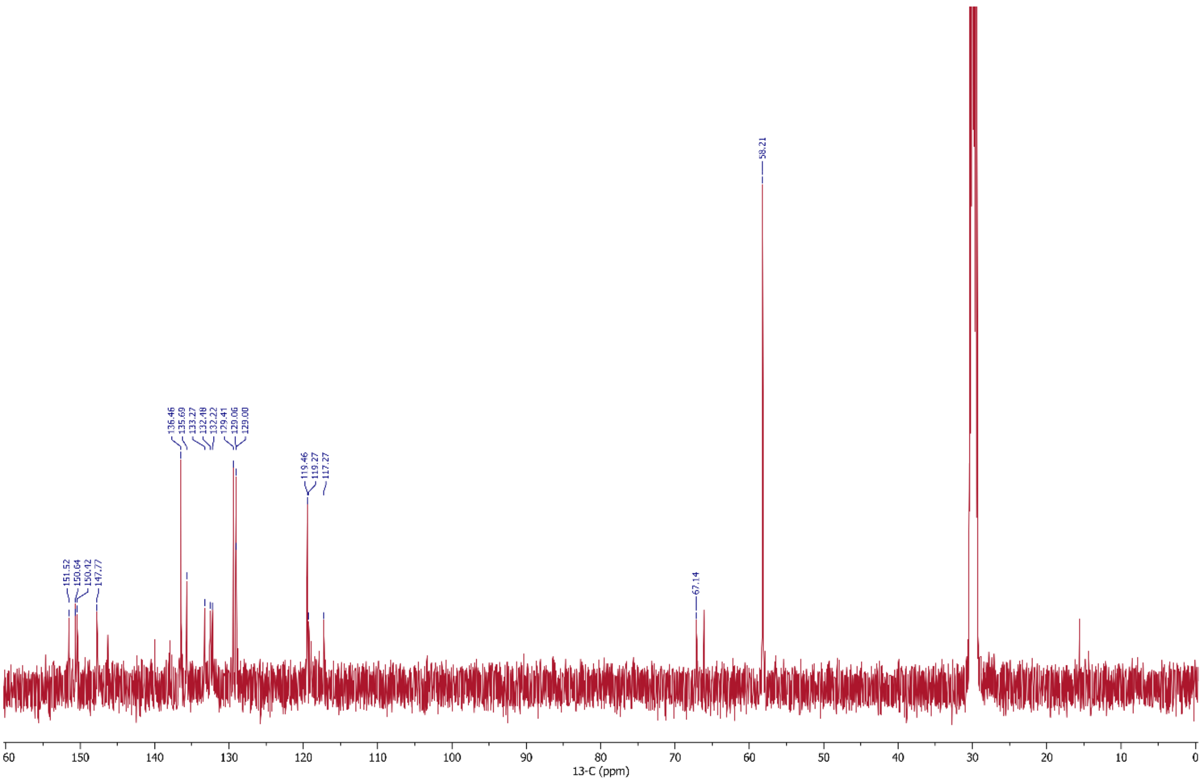

Figure S19. ^{13}C-NMR (500 MHz, 298 K, acetone-d_6) spectrum of ***p*-TMA-Cbz**.

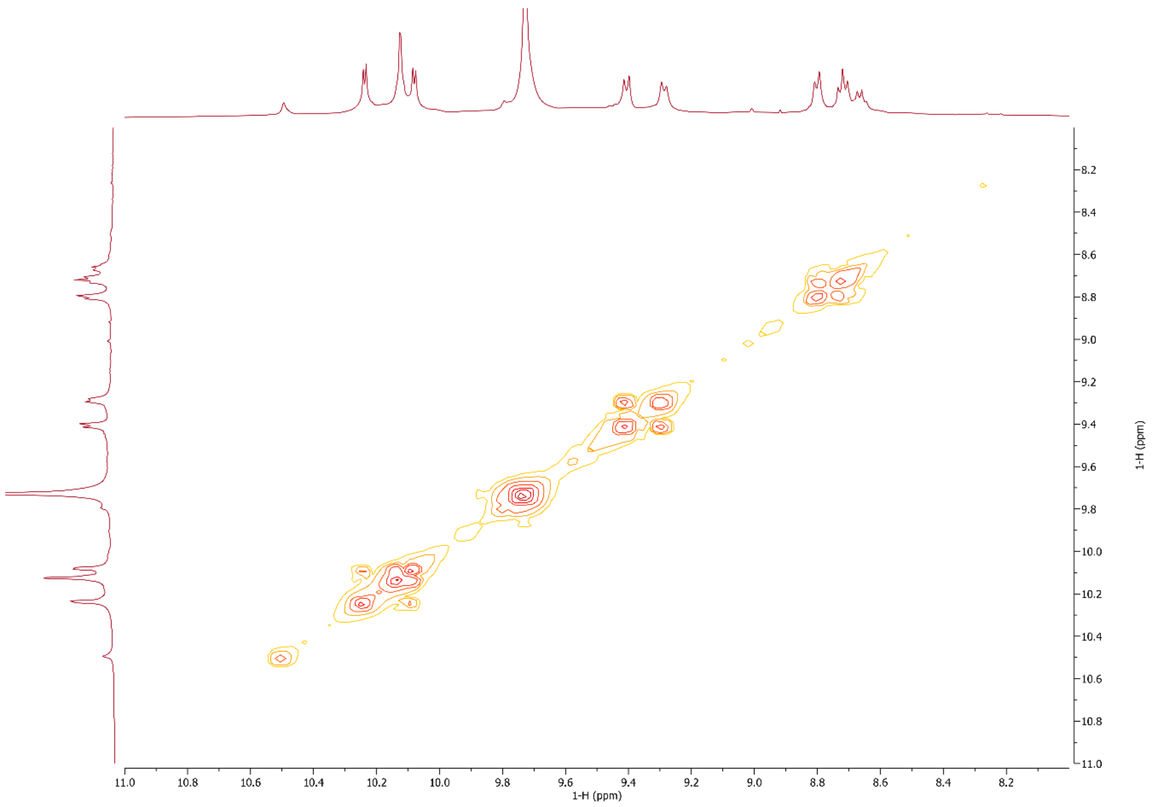

Figure S20. ^{1}H-^{1}H-COSY NMR (500 MHz, 298 K, acetone-d$_6$) spectrum of ***p*-TMA-Cbz**.

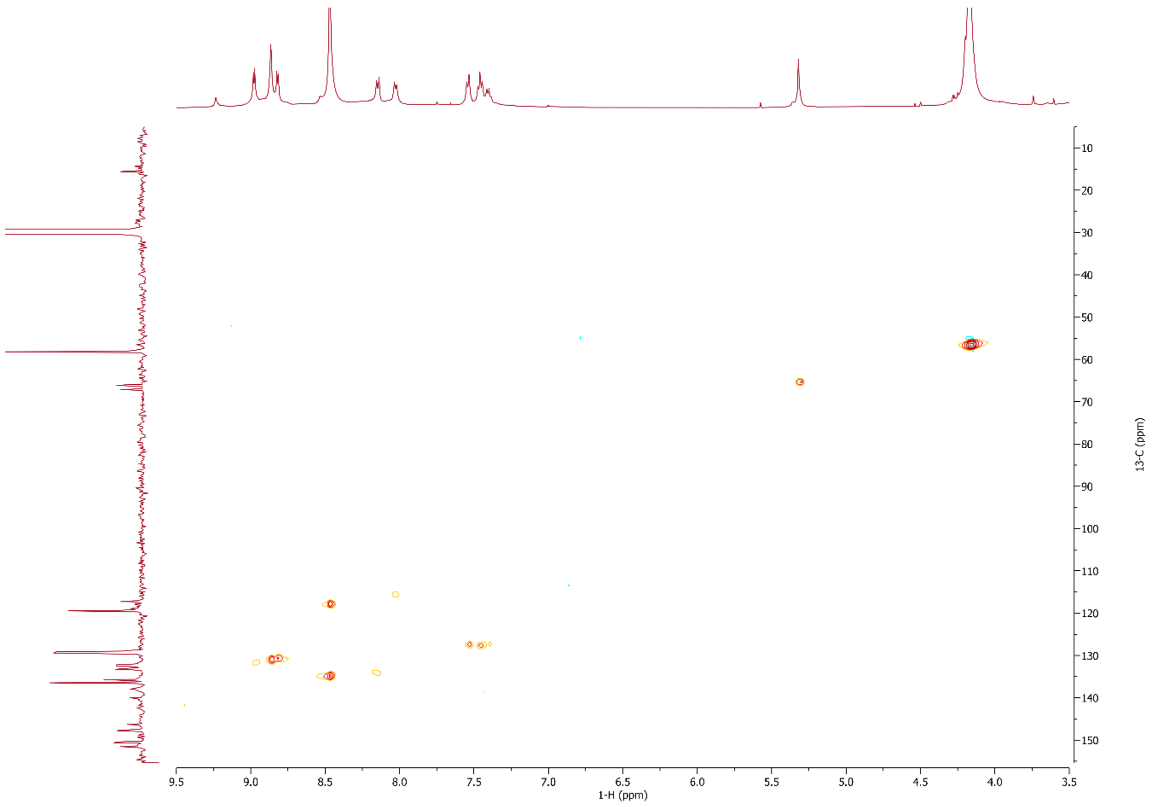

Figure S21. ^{1}H-^{13}C-HSQC NMR (500 MHz, 298 K, acetone-d$_6$) spectrum of ***p*-TMA-Cbz**.

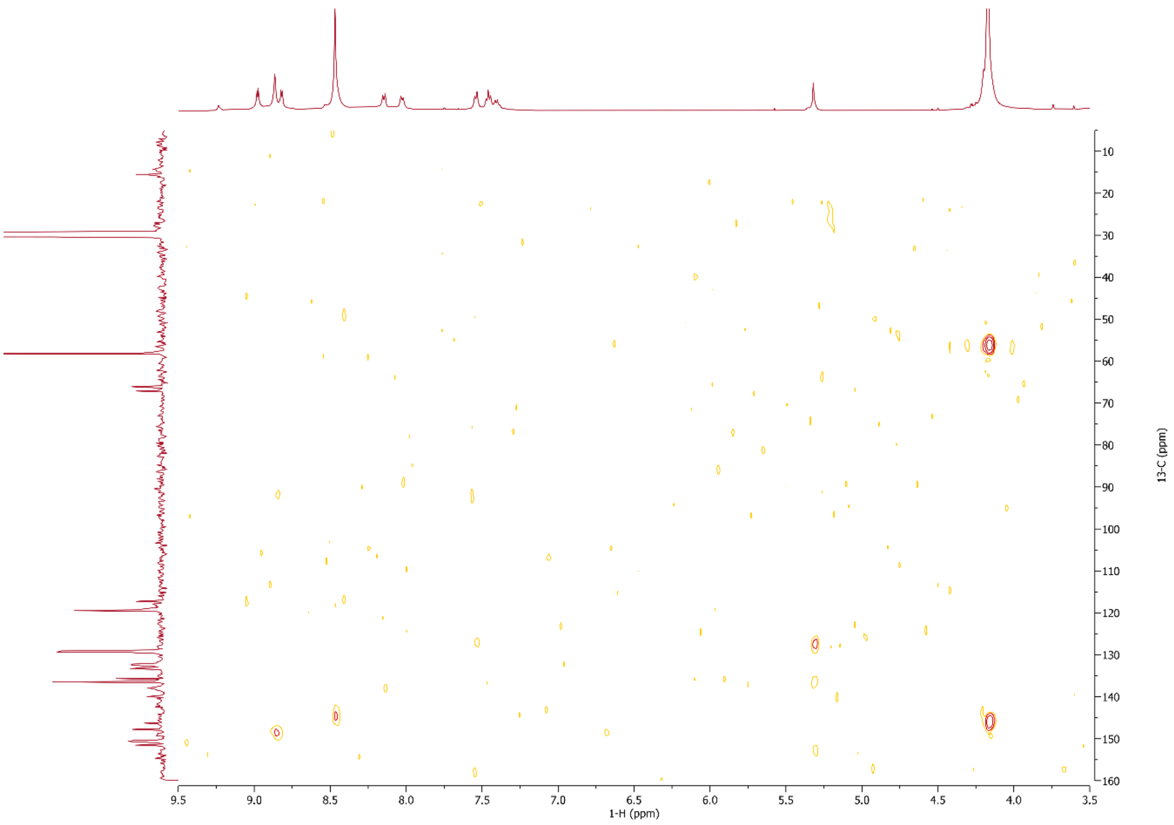

Figure S22. ^{1}H-^{13}C-HMBC NMR (500 MHz, 298 K, acetone-d_6) spectrum of ***p*-TMA-Cbz.**

3. Electrochemical characterization

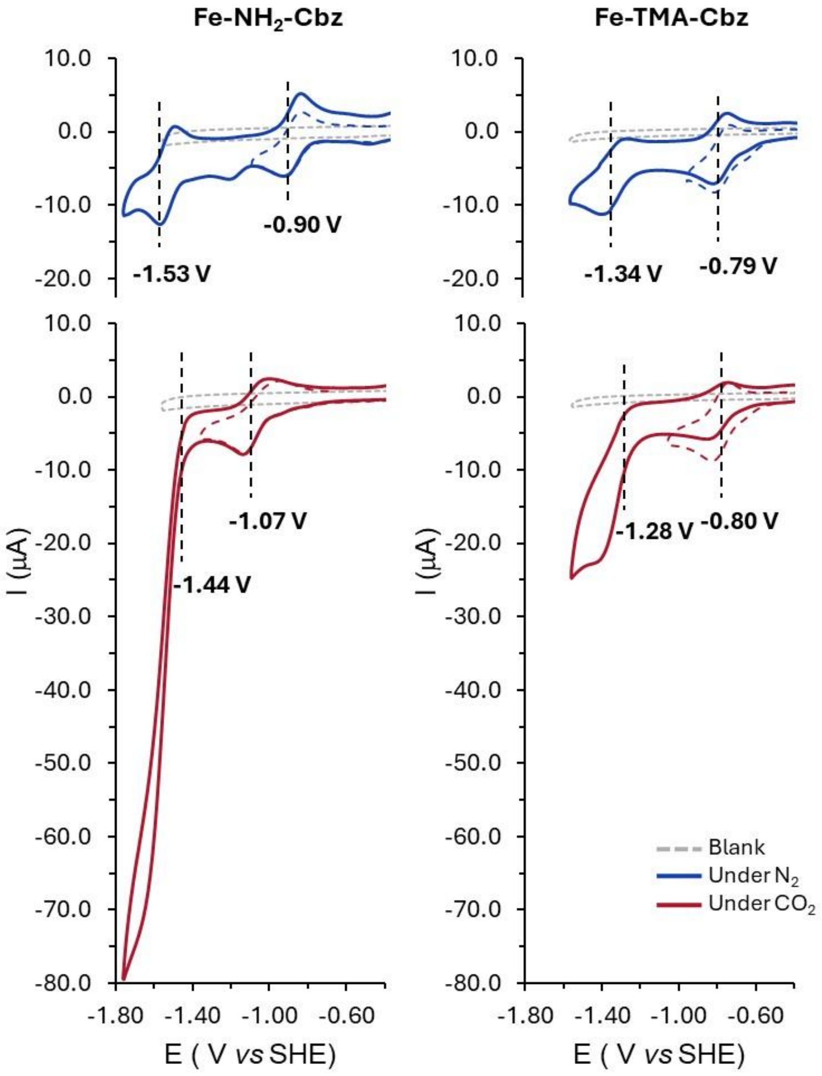

Figure S23. Cyclic voltammetry of a) **Fe-*p*-NH$_2$-Cbz** and b) **Fe-*p*-TMA-Cbz** 1 mM in DMF with 0.1 M of tetrabutylammonium hexafluorophosphate (*n*-Bu_4NPF_6) as electrolyte and 0.1 M H_2O under N_2 and CO_2 atmosphere.

4. Photocatalytic studies

Table S1. Photocatalytic CO_2R control reactions. Reported TON after 48 h.

Entry	Catalyst	Deviation from standard conditions	TON			TON[a]		
			H_2	CO	CH_4	H_2	CO	CH_4
1	No CO_2R catalyst	No CO_2R catalyst	n.d.	n.d.	n.d.	n.d.	n.d.	n.d.
2	**Fe-*p*-TMA-Cbz**	No PS	n.d.	n.d.	n.d.	n.d.	n.d.	n.d.
3	**Fe-*p*-TMA-Cbz**	No light	n.d.	n.d.	n.d.	n.d.	n.d.	n.d.
4	**Fe-*p*-TMA-Cbz**	In Ar atmosphere	n.d.	n.d.	n.d.	n.d.	n.d.	n.d.
5	**Fe-*p*-TMA-Cbz**	No TFE	n.d.	11±1	7±2	n.d.	0.1±0.01	0.1±0.02
6	**Fe-*p*-TMA-Cbz**	No SED	n.d.	31±2	8±1	n.d.	0.3±0.02	0.1±0.01
7	**Fe-*p*-TMA-Cbz**	No TFE no SED	n.d.	29±5	7±1	n.d.	0.3±0.05	0.1±0.01
8	**Fe-*p*-NH_2-Cbz**	No TFE	20±4	9±2	6±2	0.2±0.04	0.1±0.02	0.1±0.02
9	**Fe-*p*-NH_2-Cbz**	No SED	n.d.	39±6	6±1	n.d.	0.4±0.1	0.1±0.01
10	**Fe-*p*-NH_2-Cbz**	No TFE no SED	n.d.	33±4	4±1	n.d.	0.3±0.04	0.04±0.01

Standard photocatalytic conditions: CO_2R catalyst (0.01 mM), Phenox (1 mM), in DMF and TFE (0.1 M) as solvent mixture, using TEA (0.1 M) as sacrificial electron donor, reaction volume 3 mL, under visible light irradiation (447 nm), 25 °C, and under CO_2 atmosphere. [a]TON calculated regarding Phenox.

5. $^{13}CO_2$ isotopic labelling studies

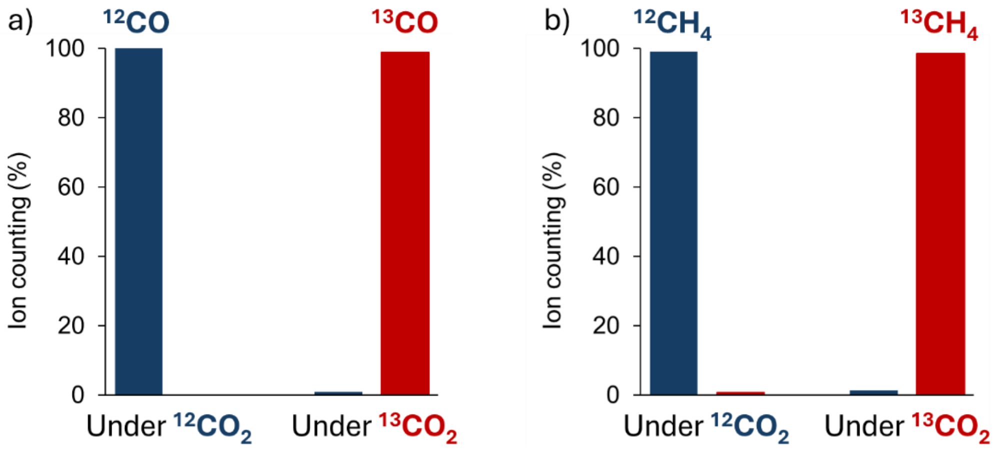

Figure S24. $^{13}CO_2$ isotopic labelling photocatalytic studies with complex **Fe–*p*–TMA–Cbz** (0.01 mM), Phenox (1 mM), in DMF and TFE (0.1 M), using TEA (0.1 M) as sacrificial electron donor, reaction volume 6 mL, under visible light irradiation (447 nm), at 25 °C, under unlabeled CO_2 or $^{13}CO_2$ atmosphere for the obtained CO (a) and CH_4 (b) products.

6. Determination of the Quantum Yield

Photodiode measurements were used to determine the incident photons by comparison with previously established actinometric values for the same photoreactor.[1-2, 7] The amount of incident photons was found to be $1.4\cdot10^{-3}$ mmol·hυ/s ($8.72\cdot10^{17}$ photons/s). Quantum yields for each product were calculated using the following expressions:

$$\Phi_{CH_4}(\%) = \frac{n_{CH_4}\cdot 8}{n_{photons}}\cdot 100$$

$$\Phi_{H_2}(\%) = \frac{n_{H_2}\cdot 2}{n_{photons}}\cdot 100$$

$$\Phi_{CO}(\%) = \frac{n_{CO}\cdot 2}{n_{photons}}\cdot 100$$

A solar-to-fuel quantum yield is defined as:

$$\Phi_{StF}(\%) = \sum \Phi_i$$

The obtained values are presented in the following table.

Table S2. Quantum yields per product and solar-to-fuel for the catalysts studied in this work.

Catalyst	mmol CH_4/s	mmol CO/s	mmol H_2/s	Φ_{CH_4} (%)	Φ_{CO} (%)	Φ_{H_2} (%)	Φ_{StF} (%)
**Fe-*p*-TMA**	$9.55\cdot10^{-10}$	$2.08\cdot10^{-9}$	0	0.0005	0.0003	0	0.0008
**Fe-*p*-NH2-Cbz**	$5.21\cdot10^{-10}$	$1.75\cdot10^{-9}$	$4.08\cdot10^{-9}$	0.0003	0.0002	0.0006	0.0011
**Fe-*p*-TMA-Cbz**	$8.68\cdot10^{-10}$	$2.03\cdot10^{-9}$	$1.04\cdot10^{-9}$	0.0005	0.0003	0.0002	0.0010

7. Reported catalysts for CO_2R to CH_4

Table S3. Previously reported electrocatalysts for CO_2R to CH_4, including faradaic efficiencies and experimental details.

**Reference**	**Catalyst**	**Faradaic efficiency for CH_4**	**Working electrode characteristics**	**Experimental conditions**	**CPE potential (V vs SHE)**
Koper *et al.*[8] (*Nat Commun*, 2015)	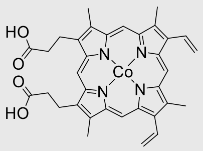	2 %	Catalyst immobilized by dip coating with a 500 mM solution in borate buffer onto a pyrolytic graphite working electrode	Aqueous perchlorate buffer 100 mM, pH 1	-1.6 (-1.2 V vs RHE)
Wang, Brudwig, *et al.*[9] (*JACS*, 2016)	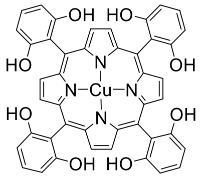	27 %	Catalyst immobilized by drop-casting onto carbon fiber paper	Aqueous 500 mM $KHCO_3$	-1.4 (-1.0 V vs RHE)
Artero, Dey, Duboc, *et al.*[10] (*ACS Energy Lett*, 2020)	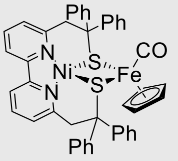	12 %	Catalyst immobilized by drop-casting with a 0.5 mM CH2Cl2 solution onto edge plane graphite working electrode	Aqueous phosphate buffer 100 mM, 3 h, pH 4	-1.3
Ertem, Grice, Angeles-Boza, *et al.*[11] (*Inorg. Chem.*, 2021)	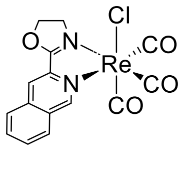	33 ± 3 %	Reticulated vitreous carbon working electrode	TFE 0.1 mM in acetonitrile, 2 h	-2.1 (-2.5 V vs Fc)
Ertem, Grice, Angeles-Boza, *et al.*[11] (*Inorg. Chem.*, 2021)	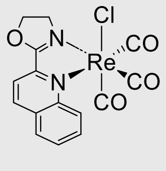	34 ± 1%	Reticulated vitreous carbon working electrode	TFE 0.1 mM in acetonitrile, 2 h	-2.1 (-2.5 V vs Fc)
Nielsen, Yang, *et al.*[12] (*JACS*, 2025)	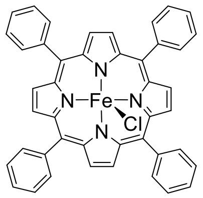 1 mM	86 ± 5 %	Carbon fiber working electrode	THF, 60 mM H_2O as proton source, 0.1 M n-Bu_4NPF_6, 1,3-bis(2,6-diisopropylphenyl)imidazol-2-ylidene 20mM, 63 h (reduction of the Sorbed-CO_2 on the DPICx molecule)	-1.95 (-2.35 V vs Fc)

Table S4. Previously reported photocatalysts for CO_2R to CH_4, including experimental details and quantum yield.

Reference	Catalyst	TON (Selectivity) CH_4	Sacrificial reagents	Experimental conditions	Energy source	Quantum yield
Robert, Bonin, *et al.*[13] (*Nature*, 2017)	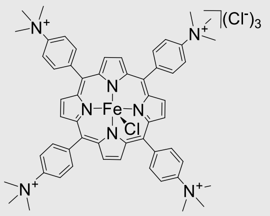 2 µM	31 (12 %)	TEA 50 mM	Ir(ppy)$_3$ 200 µM, in MeCN, 47 h	Newport LCS-100 solar simulator 1 Sun irradiance, with filters for $\lambda > 420$ nm	0.18 % (CO-to-CH_4)
Robert, Bonin, *et al.*[13] (*Nature*, 2017)	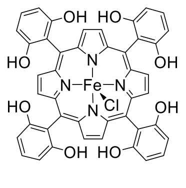 2 µM	26 (14 %)	TEA 50 mM	Ir(ppy)$_3$ 200 µM, in MeCN, 47 h	Newport LCS-100 solar simulator 1 Sun irradiance, with filters for $\lambda > 420$ nm	Not reported
Robert, Miyake, *et al.*[14] (JACS, 2018)	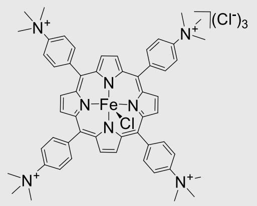 10 µM	71 (75 %)	TEA 100 mM	Phenox 1000 µM, TFE 100 mM, in DMF, 47 h	Newport LCS-100 solar simulator 1 Sun irradiance, with filters for $\lambda > 435$ nm	0.47 % (CO-to-CH_4)
**This work**	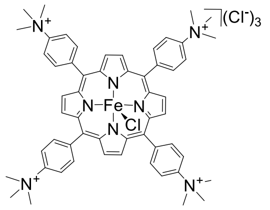 10 µM	11 ± 2 (32 %)	TEA 100 mM	Phenox 1000 µM, TFE 100 mM, in DMF, 96 h	Treellum Technologies® photoreactor, λ = (447 ± 20) nm, 1030 mW at 700 mA	0.0006 % (CO_2-to-CH_4)
**This work**	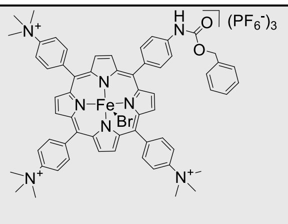 10 µM	10 ± 3 (22 %)	TEA 100 mM	Phenox 1000 µM, TFE 100 mM, in DMF, 96 h	Treellum Technologies® photoreactor, λ = (447 ± 20) nm, 1030 mW at 700 mA	0.0005 % (CO_2-to-CH_4)
**This work**	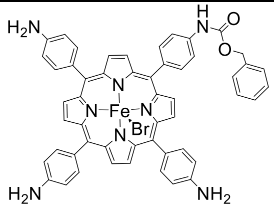 10 µM	6 ± 1 (8 %)	TEA 100 mM	Phenox 1000 µM, TFE 100 mM, in DMF, 96 h	Treellum Technologies® photoreactor, λ = (447 ± 20) nm, 1030 mW at 700 mA	0.0003 % (CO_2-to-CH_4)

8. References

1. Casadevall, C.; Pascual, D.; Aragon, J.; Call, A.; Casitas, A.; Casademont-Reig, I.; Lloret-Fillol, J., *Chem Sci* **2022**, 13 (15), 4270-4282.
2. Casadevall, C.; Aragón, J.; Cañellas, S.; Pericàs, M. A.; Lloret-Fillol, J.; Caldentey, X., American Chemical Society: 2022; Vol. 1419, pp 145-165.
3. Lloret-Fillol, J.; Casadevall Serrano, C.; Leon, J. L.; Call Quintana, A.; Casitas Montero, A.; Pla, J. J.; Perez Hernandez, J.; Caldentey Frontera, F. X. A photoreactor providing high light intensity accelerating the reactions and improving reproducibility of the reactions by temperature and light intensity control as well as calibration in a high throughput exptl. environment. EP3409352A1, 2018.
4. Call, A.; Casadevall, C.; Acuna-Pares, F.; Casitas, A.; Lloret-Fillol, J., *Chem Sci* **2017**, 8 (7), 4739-4749.
5. Costentin, C.; Robert, M.; Savéant, J. M.; Tatin, A., *Proc. Natl. Acad. Sci. U. S. A.* **2015**, 112, 6882.
6. Boucher, L. J.; Katz, J. J., *J. Am. Chem. Soc.* **1967**, 89 (6), 1340-1345.
7. Call, A.; Franco, F.; Kandoth, N.; Fernandez, S.; Gonzalez-Bejar, M.; Perez-Prieto, J.; Luis, J. M.; Lloret-Fillol, J., *Chem. Sci.* **2018**, 9 (9), 2609-2619.
8. Shen, J.; Kortlever, R.; Kas, R.; Birdja, Y. Y.; Diaz-Morales, O.; Kwon, Y.; Ledezma-Yanez, I.; Schouten, K. J. P.; Mul, G.; Koper, M. T. M., *Nat. Commun.* **2015**, 6, 8177.
9. Weng, Z.; Jiang, J.; Wu, Y.; Wu, Z.; Guo, X.; Materna, K. L.; Liu, W.; Batista, V. S.; Brudvig, G. W.; Wang, H., *J. Am. Chem. Soc.* **2016**, 138, 8076.
10. Ahmed, M. E.; Adam, S.; Saha, D.; Fize, J.; Artero, V.; Dey, A.; Duboc, C., *ACS Energy Lett.* **2020**, 5 (12), 3837-3842.
11. Nganga, J. K.; Wolf, L. M.; Mullick, K.; Reinheimer, E.; Saucedo, C.; Wilson, M. E.; Grice, K. A.; Ertem, M. Z.; Angeles-Boza, A. M., *Inorg. Chem.* **2021**, 60 (6), 3572-3584.
12. Stanley, J. S.; Pauker, H. N.; Kuker, E.; Dong, V.; Nielsen, R. J.; Yang, J. Y., *J. Am. Chem. Soc.* **2025**, 147 (19), 16099-16106.
13. Rao, H.; Schmidt, L. C.; Bonin, J.; Robert, M., *Nature* **2017**, 548, 74.
14. Rao, H.; Lim, C.-H.; Bonin, J.; Miyake, G. M.; Robert, M., *J. Am. Chem. Soc.* **2018**, 140 (51), 17830-17834.
